# Mkt1 is required for RNAi-mediated silencing and establishment of heterochromatin in fission yeast

**DOI:** 10.1093/nar/gkz1157

**Published:** 2019-12-11

**Authors:** Francesca Taglini, Elliott Chapman, Rob van Nues, Emmanuelle Theron, Elizabeth H Bayne

**Affiliations:** Institute of Cell Biology, School of Biological Sciences, The University of Edinburgh, Edinburgh, UK

## Abstract

Constitutive domains of repressive heterochromatin are maintained within the fission yeast genome through self-reinforcing mechanisms involving histone methylation and small RNAs. Non-coding RNAs generated from heterochromatic regions are processed into small RNAs by the RNA interference pathway, and are subject to silencing through both transcriptional and post-transcriptional mechanisms. While the pathways involved in maintenance of the repressive heterochromatin state are reasonably well understood, less is known about the requirements for its establishment. Here, we describe a novel role for the post-transcriptional regulatory factor Mkt1 in establishment of heterochromatin at pericentromeres in fission yeast. Loss of Mkt1 does not affect maintenance of existing heterochromatin, but does affect its recovery following depletion, as well as *de novo* establishment of heterochromatin on a mini-chromosome. Pathway dissection revealed that Mkt1 is required for RNAi-mediated post-transcriptional silencing, downstream of small RNA production. Mkt1 physically associates with pericentromeric transcripts, and is additionally required for maintenance of silencing and heterochromatin at centromeres when transcriptional silencing is impaired. Our findings provide new insight into the mechanism of RNAi-mediated post-transcriptional silencing in fission yeast, and unveil an important role for post-transcriptional silencing in establishment of heterochromatin that is dispensable when full transcriptional silencing is imposed.

## INTRODUCTION

Heterochromatin is a key structural and regulatory component of eukaryotic chromosomes. In contrast to euchromatic regions that are permissive for gene expression, domains of heterochromatin are generally repressive. They are characterized by low levels of histone acetylation, and in most eukaryotes by high levels of methylation of histone H3 at lysine 9 (H3K9me), which provides binding sites for chromodomain proteins including HP1 (heterochromatin protein 1) that promotes chromatin compaction and transcriptional silencing (1). Large domains of constitutive heterochromatin are typically found at centromeres and telomeres, functioning to silence repetitive elements, regulate recombination, and ensure proper chromosome segregation. In addition, facultative heterochromatin domains contribute to the control of developmentally regulated loci, and play an important role in locking in differentiated cell states (2). Assembly of heterochromatin is thought to proceed through three distinct phases of nucleation, spreading and maintenance (3,4). Proper spatial regulation of heterochromatin requires mechanisms for accurate targeting and confinement of chromatin modifiers to specific DNA sequences, and increasing evidence points to widespread roles for non-coding RNAs in this process (5,6).

The fission yeast *Schizosaccharomyces pombe* has proved a fruitful model for dissecting mechanisms of heterochromatin assembly, with core structural and regulatory features conserved in higher eukaryotes. Fission yeast centromeres are flanked by outer repeat sequences that are assembled in heterochromatin, and establishment and maintenance of this pericentromeric heterochromatin is dependent on the RNA interference (RNAi) pathway. Although heterochromatic, the pericentromeric repeat sequences are transcribed by RNA polymerase II (RNAPII), giving rise to double-stranded RNA (dsRNA) that is processed into short interfering RNAs (siRNAs) by the Dicer ribonuclease Dcr1 (7–9). These siRNAs are bound by the Argonaute protein Ago1 as part of the RNA-induced transcriptional silencing (RITS) complex, guiding it to complementary nascent transcripts (10). Through the adaptor protein Stc1 (11,12), chromatin-associated RITS recruits the sole H3K9 methyltransferase Clr4, as part of the Clr4 complex (CLRC), leading to H3K9 methylation in cognate chromatin (13). The resulting H3K9 methyl mark facilitates binding of chromodomain proteins including Swi6 (the fission yeast HP1), Chp1 (a subunit of RITS), and Clr4, thereby creating a positive feedback loop for further recruitment of both RNAi and chromatin modification factors (13–15). The activity of histone deacetylases including Sir2 and Clr3 is also important for the spreading and maintenance of heterochromatin domains (4,16). Whereas at centromeres RNAi is important for both establishment and maintenance of heterochromatin, other constitutive heterochromatin domains at telomeres and the silent mating-type locus depend on RNAi only for establishment but not for maintenance, due to the presence of alternative pathways involving DNA-binding proteins acting redundantly with RNAi (17–19).

Heterochromatic silencing involves repression at both transcriptional and post-transcriptional levels. In fission yeast, RNAi not only promotes recruitment of factors mediating transcriptional repression, but also contributes to silencing of heterochromatic transcripts independently of H3K9 methylation through co-transcriptional RNA processing (8). In addition, other RNA processing factors also contribute to heterochromatic silencing. At centromeres, there is evidence of parallel RNAi-independent pathways promoting heterochromatin assembly involving the nuclear exosome component Rrp6 (20), as well as Dhp1, a conserved 5′-3′ exoribonuclease involved in transcription termination and RNA quality control (21,22). In addition, assembly of a subset of facultative heterochromatin domains, so-called ‘HOODs’, has been found to depend on both the RNAi machinery, and the RNA processing and surveillance complex MTREC (Mtl1-Red1 core) and associated factors (23–25). HOODs are found at discrete genomic regions including transposons and developmentally regulated genes, and are formed in particular conditions, including in the absence of functional Rrp6. The MTREC complex is also required for formation of another class of facultative heterochromatin domains, so-called heterochromatin islands, found at meiotic genes that are silenced during vegetative growth. In contrast to HOODs, heterochromatin islands are RNAi-independent, and instead locus-specific targeting involves the sequence-specific RNA-binding protein Mmi1 (26–28). Although H3K9 methylation is present, silencing at heterochromatin islands depends primarily on post-transcriptional mechanisms (29), and in most cases the relationship between post-transcriptional regulation and heterochromatin assembly remains unclear.

Recent studies have confirmed that heterochromatin in fission yeast has the potential for epigenetic inheritance: once established at a locus, H3K9 methylation can be propagated through cell division independently of the original targeting signal or any specific DNA sequence (30,31). A prediction from these properties is that there may be factors required for establishment of heterochromatin, but dispensable for maintenance. Indeed, a recently identified example of such a factor is Triman, a 3′-5′ exonuclease involved in siRNA biogenesis and required for establishment but not maintenance of pericentromeric heterochromatin (32). However, thus far, studies aimed at identification of heterochromatin assembly factors have largely focused on isolation of mutants disrupting maintenance of existing constitutive heterochromatin domains, meaning that factors with more pronounced roles in the establishment phase of heterochromatin assembly may have been overlooked. Here, we identify Mkt1 as a novel factor required for RNAi-mediated silencing in fission yeast. Mkt1 is a post-transcriptional regulatory factor that is not required for maintenance of existing pericentromeric heterochromatin, but is required for efficient re-establishment of pericentromeric H3K9 methylation following its depletion, as well as for efficient *de novo* establishment of heterochromatin on a minichromosome. Outwith constitutive heterochromatin domains, Mkt1 also plays roles in maintenance of silencing and H3K9 methylation in certain cases, including some HOODs. Mkt1 physically associates with pericentromeric transcripts, and becomes important for maintenance of silencing and heterochromatin at pericentromeres when transcriptional silencing is impaired. Our findings reveal a role for Mkt1 in RNAi-mediated post-transcriptional silencing in fission yeast, and indicate that this post-transcriptional silencing operates redundantly with transcriptional silencing for maintenance of pericentromeric heterochromatin, but is required for its efficient establishment.

## MATERIALS AND METHODS

### Yeast strains and genetic manipulation

The genome-wide genetic screen was performed using version 2 of the Bioneer haploid deletion library (33); further details are provided in the Supplementary Data. All other fission yeast strains used in this study are listed in Supplementary Table S1. Standard procedures were used for growth and genetic manipulations. Genomic integrations for gene deletion and epitope-tagging were achieved by homologous recombination using PCR-based modules consisting of a resistance cassette flanked by sequences homologous to the target locus. To generate *mkt1^+^* point mutants, the *mkt1^+^* gene was cloned in plasmid pJET, and mutagenized using the Quikchange Lightning Site-Directed Mutagenesis Kit (Agilent) according to the manufacturer's instructions. The mutated ORFs were PCR amplified and reintegrated at the endogenous locus. The minichromosome establishment assay was carried out as previously described (4).

### RNA analysis

Total RNA was extracted from 1 × 10^7^ cells in exponential growth phase using the Masterpure Yeast RNA Purification Kit (Epicentre) according to the manufacturer's instructions. For RT-qPCR analysis, 1 μg of total RNA was treated with TURBO DNase (Ambion) for 1 h at 37°C, then reverse transcribed using random hexamers (Roche) and Superscript III reverse transcriptase (Invitrogen) according to the manufacturer's instructions. cDNA was quantified by qPCR using LightCycler 480 SYBR Green (Roche) and primers listed in Supplementary Table S2. In all cases, histograms represent three biological replicates and error bars represent one S.D. For RNA-seq analysis, libraries were prepared using Illumina TruSeq stranded mRNA library preparation kit according to manufacturer's instructions, then pooled and paired-end sequenced on a HiSeq4000. Raw reads were filtered for quality and adapter using cutadapt (v1.8.3). Trimmed reads were then aligned to the *S. pombe* reference genome (ASM294v2.30) using STAR v2.5. Read counts were obtained using HTSeq v.0.6.1 with mode ‘union’, and differential gene expression then performed using edgeR (version 3.12.0). Genes with <20 reads were discarded from the analysis, and those with a corrected *P*-value < 0.05 were retained. For visualisation in IGV, bigwig coverage files were generated using RPKM normalisation to account for between-samples differences in sequencing depth.

Small RNAs were extracted by resuspending 5 × 10*^8^* cells in 50 mM Tris–HCl pH 7.5, 10 mM EDTA pH 8, 100 mM NaCl, 1% SDS, adding equal volumes of phenol:chloroform 5:1 and acid washed beads, and vortexing for 30 min at 4°C. The soluble fraction was extracted with phenol/chloroform and long RNAs precipitated with 10% polyethylene glycol 8000 and 0.5 M NaCl on ice for 30 min. The supernatant was recovered and small RNAs precipitated with ethanol overnight at −20°C. Northern analysis of small RNAs was performed as described previously (34). Briefly, RNA samples were run on a 12% polyacrylamide gel, electrophoretically transferred onto Hybond-NX (Amersham) and crosslinked by incubation at 55°C for 2 h with a 0.16 M carbodiimide, 1-ethyl-3-(3-dimethylaminopropyl) carbodiimide (EDC) solution. Membranes were probed with 5′ end radiolabelled oligonucleotides listed in Supplementary Table S2.

### Immunoaffinity purification

ChIP and DRIP experiments were performed essentially as described previously (35). Briefly, 2.5 × 10*^8^* cells per IP were fixed in 1% formaldehyde for 15 min at room temperature. Cells were lysed using a bead beater (Biospec products) and sonicated using a Bioruptor (Diagenode) for a total of 20 min (30 s on/30 s off on ‘high’ power). Immunoprecipitation was then performed overnight at 4°C, using 1 μl per IP of monoclonal anti-H3K9me2 (5.1.1 (36)), or polyclonal anti-H3K9me3 (C15410193, Diagenode), for ChIP, or 1 μl of monoclonal S9.6 anti-DNA-RNA hybrid (MABE1095, MerckMilipore) for DRIP. Immunoprecipitated DNA was recovered using Chelex-100 resin (BioRad), and quantified by qPCR using LightCycler 480 SYBR Green (Roche) and primers listed in Supplementary Table S2. Relative enrichments were calculated as the ratio of product of interest to control product (*act1^+^*) in IP over input. In all cases, histograms represent three biological replicates and error bars represent one S.D.

Immunoaffinity purifications for mass-spec analysis were performed essentially as described previously (37). Briefly, *S. pombe* cultures were grown to a cell density of 10^8^ cells/ml in 4× concentrated YES media. For each sample, 5 g of cells, milled in solid phase, were used. Cell powder was resuspended in lysis buffer (50 mM HEPES–NaOH [pH 7.5], 150 mM NaCl, 1 mM MgCl_2_, 0.1% NP-40, 5 mM DTT, 0.5 mM PMSF, 1× EDTA-free protease inhibitor cocktail [Roche]), and immunoprecipitations performed using Dynabeads coupled to anti-FLAG antibody (Sigma, F3165) for 60 min at 4°C. The immunoprecipitated material was treated with 500 U of Benzonase, washed with lysis buffer, and resuspended in 8 M urea. Proteins were then reduced in 10 mM dithiothreitol for 30 min at room temperature, alkylated in 55 mM iodoacetamide for another 30 min in the dark, and then digested with LysC (1;50 μg of protein) (Wako Chemicals, Japan) for 4 h. Samples were then further diluted with 50 mM ammonium bicarbonate to achieve urea concentration <2 M prior to overnight digestion with trypsin (1;50 μg of protein) (Pierce Scientific) at room temperature. Following digestion, samples were acidified with 10% TFA to pH <2.5 and spun onto StageTips as described previously (38). Peptides were eluted in 40 μl of 80% acetonitrile in 0.1% TFA and concentrated down to 1 μl by vacuum centrifugation (Concentrator 5301, Eppendorf). Samples were then diluted to 5 μl in 0.1% TFA and injected for LC–MS/MS analysis.

RNA-IPs were performed as above but with the addition of RNasin ribonuclease inhibitors (1:200, Promega) to the lysis buffer, and excluding Benzonase treatment. After washes, immunoprecipitated material was resuspended in RNA extraction buffer (25 mM Tris–HCl pH 7.5, 5 mM EDTA pH 8, 50 mM NaCl, 0.5% SDS) with 200 ng/ml proteinase K and incubated at 37°C for 2 h. RNA was extracted with phenol:chloroform, precipitated with ethanol and 1 μl glycogen, and analysed by qRT-PCR.

### CRAC

1 × 10^7^ cells grown to exponential phase in PMG minus tryptophan medium were UV-irradiated in a Vari-X-linker for 42.5 s and processed to obtain cDNA libraries as described previously (39). Libraries were paired-end sequenced on a HiSeq4000. Raw fastq files were pre-processed using the pyCRAC package (pypi.org/project/pyCRAC/). Reads were demultiplexed (script pyBarcodeFilter.py) and adapter sequences were removed with Flexbar (github.com/seqan/flexbar). PCR-duplicates (pyFastqDuplicateRemover.py) and tRNA reads (STAR version 2.7.1.a; github.com/alexdobin/STAR/) were filtered out before reads were mapped to the *S. pombe* genome (ASM294v2.30) using novoalign (version 3.09.02). Mapped reads were counted (pyReadCounters.py, using options ‘–mutations = delsonly –blocks’) and for presentation purposes data from two biological replicates were merged prior to generation of bedgraph files (pyGTF2bedGraph.py, with options ‘–count –permillion’), or metagene distribution plots (pyBinCollector.py, using the indicated number of bins). From the gene-plot, a list of mRNAs crosslinked to Mkt1 at their 3′ ends was derived (pyBinCollector.py with option ‘–binoverlap 80 100’, and pyGetGeneNamesFromGTF.py, with options ‘–attribute = gene_name –count’). Genes with fewer than ten counts in each of two biological replicates were discarded. Bedgraph files were visualized using the GenomeBrowser (pypi.org/project/GenomeBrowser).

## RESULTS

### Mkt1 is a novel factor required for RNAi-mediated silencing

**Figure 1.**
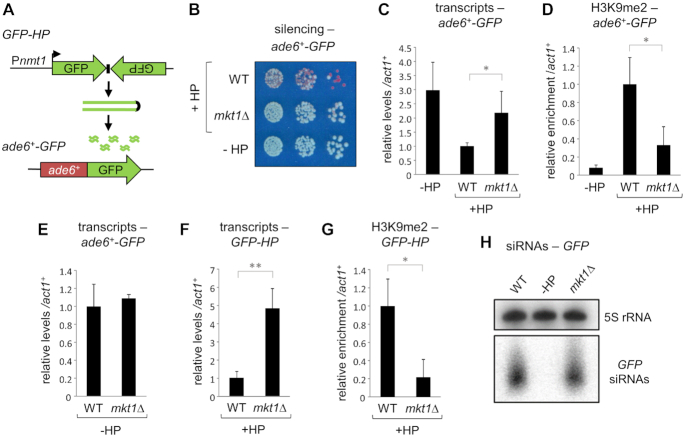
Mkt1 is required for efficient hairpin-mediated silencing and heterochromatin assembly in fission yeast. (**A**) Schematic representation of the hairpin silencing system comprising a GFP hairpin construct (*GFP-HP*) expressed under the *nmt* promoter as a source of *GFP* siRNAs, and an *ade6^+^-GFP* fusion expressed from the endogenous *ade6^+^* locus as a reporter. (**B**) Assay for silencing of the *ade6^+^-GFP* reporter gene: red colonies on low adenine media indicate silencing, and white colonies absence of silencing. (**C**) RT-qPCR analysis of *ade6^+^-GFP* transcript levels relative to *act1*^+^, normalized to wild-type. (**D**) ChIP-qPCR analysis of H3K9me2 levels at the *ade6^+^-GFP* locus relative to *act1^+^*, normalised to wild-type. (**E**) RT-qPCR analysis of *ade6^+^-GFP* transcript levels in the absence of the hairpin. (**F**) RT-qPCR analysis of *GFP-HP* transcript levels. (**G**) ChIP-qPCR analysis of H3K9me2 levels at the *GFP-HP* locus. In each case data are averages of three biological replicates and error bars represent one SD; *P < 0.05, **P < 0.01. (**H**) Northern analysis of *GFP* siRNAs (5S rRNA is a loading control).

To identify novel factors involved in RNAi-mediated silencing and heterochromatin assembly in *S. pombe*, we performed a sensitized, systematic genetic screen based on maintenance of silencing triggered by a hairpin RNA. Hairpin-derived siRNAs can act in *trans* to trigger heterochromatin assembly and silencing at an otherwise euchromatic locus; however, in comparison to that at constitutive heterochromatin domains, such hairpin-mediated silencing is generally inefficient and unstable (40,41). We reasoned that screening for factors required to maintain such unstable silencing would provide a sensitized system that could reveal additional pathway components dispensable for maintenance of more robust heterochromatin domains. We employed a tester strain bearing a previously described *trans* RNAi silencing system comprising a GFP hairpin RNA (*GFP-HP*) as a source of GFP siRNAs, and an *ade6^+^-GFP^+^* fusion gene as a reporter (Figure 1A) (41). Silencing of the reporter can be assessed via colony colour: in the absence of the GFP-HP construct *ade6^+^-GFP^+^* is expressed, resulting in white colonies on low adenine media; when the GFP-HP RNA is present it is processed into GFP siRNAs that direct silencing of the *ade6^+^-GFP^+^* locus, resulting in the appearance of red colonies (Figure 1B). In an otherwise wild-type background, a mixed population of red and white colonies is observed, reflecting the relative instability of silencing in this system. Exploiting this setup, we screened a library of ∼3000 haploid deletion strains for altered hairpin-dependent silencing of *ade6^+^-GFP^+^*. In addition to the many known RNAi/heterochromatin factors, we identified *mkt1^+^* as a novel gene required to maintain silencing of *ade6^+^-GFP^+^*, indicated by an absence of red colonies in the *mkt1Δ* strain (Figure 1B). RT-qPCR and H3K9me2 ChIP-qPCR analyses confirmed that *mkt1^+^* deletion is associated with increased accumulation of *ade6^+^-GFP^+^* transcripts, as well as loss of H3K9 methylation from the target locus (Figure 1C and D). Deletion of *mkt1^+^* does not affect the expression of *ade6^+^-GFP^+^*in the absence of the hairpin RNA (Figure 1E), confirming that the effect is specific to RNAi-mediated silencing.

While the *ade6^+^-GFP^+^* locus is subject to silencing in *trans* in this system, the GFP hairpin-derived siRNAs also induce partial silencing of the GFP-HP locus itself in *cis* (41). RT-qPCR and ChIP-qPCR analyses revealed that deletion of *mkt1^+^* also results in increased accumulation of hairpin transcripts and a reduction in H3K9me2 levels at the GFP-HP locus, confirming a requirement for Mkt1 in RNAi-mediated silencing both in *cis* and in *trans* (Figure 1F and G). The observed silencing defects were not the result of impaired production of GFP siRNAs, as northern blot analyses showed similar levels of GFP siRNAs in wild-type and *mkt1Δ* cells (Figure 1H). Together, these observations indicate that deletion of *mkt1^+^* results in a defect in RNAi-mediated silencing, downstream of siRNA accumulation.

### Mkt1 is required for establishment but not maintenance of heterochromatin at pericentromeres

**Figure 2.**
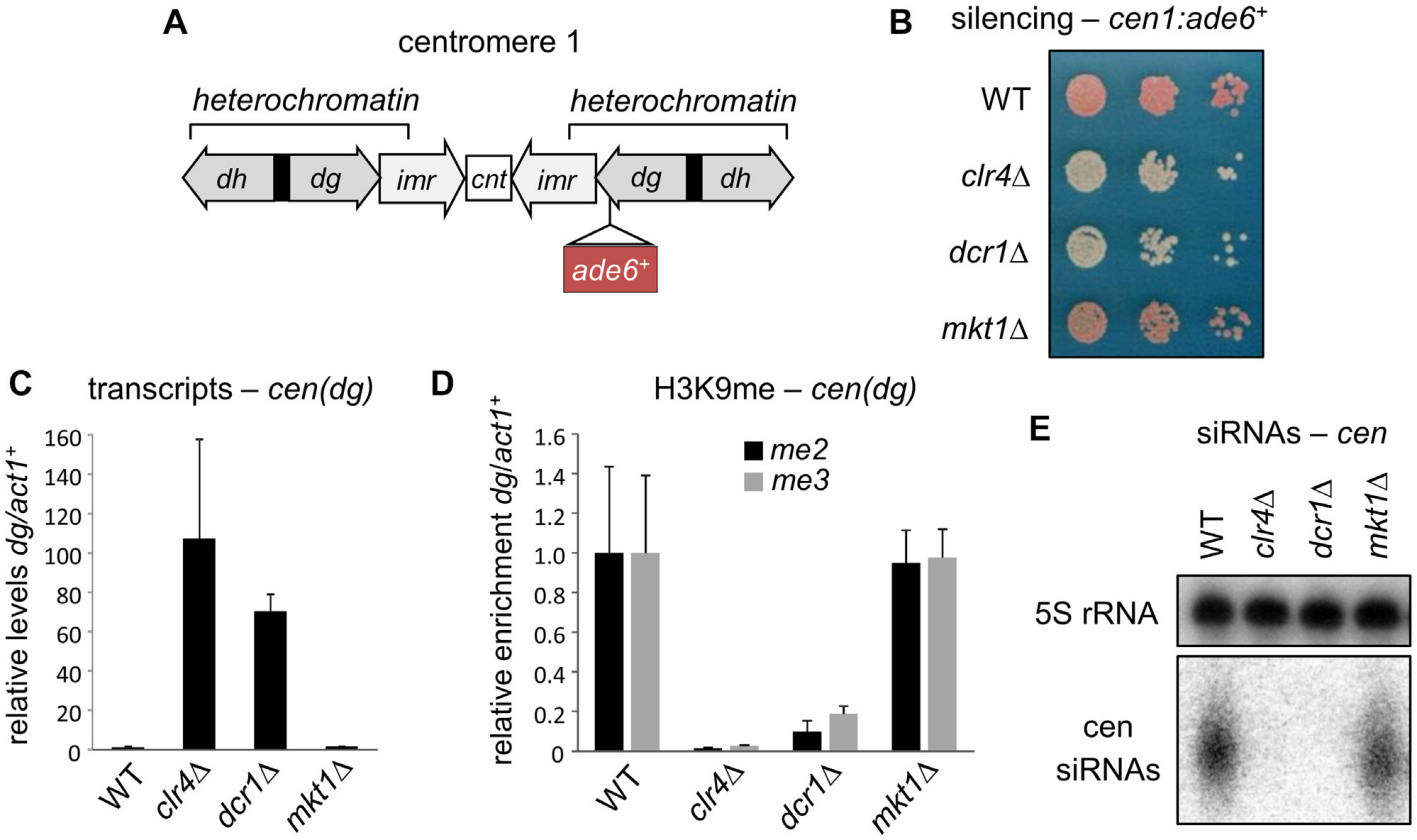
Mkt1 is not required for maintenance of silencing and heterochromatin at pericentromeres. (**A**) Schematic representation of the *cen1:ade6^+^* reporter, indicating the position of the *ade6^+^* insertion in centromere one relative to centromeric outer repeats (*dg* and *dh*), innermost repeats (*imr*) and central core (*cnt*). (**B**) Assay for silencing of the *cen1*:*ade6^+^* reporter: silencing results in red colonies on low adenine media; loss of silencing results in pink/white colonies. (**C**) RT-qPCR analysis of *cen(dg)* transcript levels relative to *act1*^+^, normalized to wild-type. (**D**) ChIP-qPCR analysis of H3K9me2 and H3K9me3 levels at *cen(dg)* relative to *act1^+^*, normalised to wild-type. (**E**) Northern analysis of centromeric siRNAs (5S rRNA is a loading control).

The endogenous RNAi-mediated silencing that occurs at centromeres operates in *cis* and is therefore conceptually similar to the *cis* silencing seen at the GFP-HP locus, but is more robust. To assess whether *mkt1^+^* is also required for silencing at centromeres, we deleted *mkt1^+^* in a strain bearing an *ade6^+^* reporter gene inserted into the heterochromatic outer repeats on the right side of centromere 1 (*cen1:ade6^+^*, Figure 2A). Wild-type cells generate red colonies on low adenine media due to silencing of the *ade6^+^* reporter, whereas deletion of genes required for silencing, such as *clr4^+^* or *dcr1^+^*, results in pink or white colonies. Surprisingly, we observed no difference in colony colour between wild-type and *mkt1Δ* cells, indicating that loss of *mkt1^+^* does not impair maintenance of pericentromeric silencing (Figure 2B). Consistent with this, in *mkt1Δ* cells we observed no accumulation of pericentromeric transcripts, and no reduction in levels of H3K9me2 at pericentromeric repeat sequences. Levels of H3K9me3, recently reported to be required for full transcriptional repression (42), were also unaffected, and there was no change in accumulation of pericentromeric siRNAs (Figure 2C–E). Other major constitutive heterochromatin domains at telomeres and the mating-type locus were similarly unaffected (Supplementary Figure S1).

**Figure 3.**
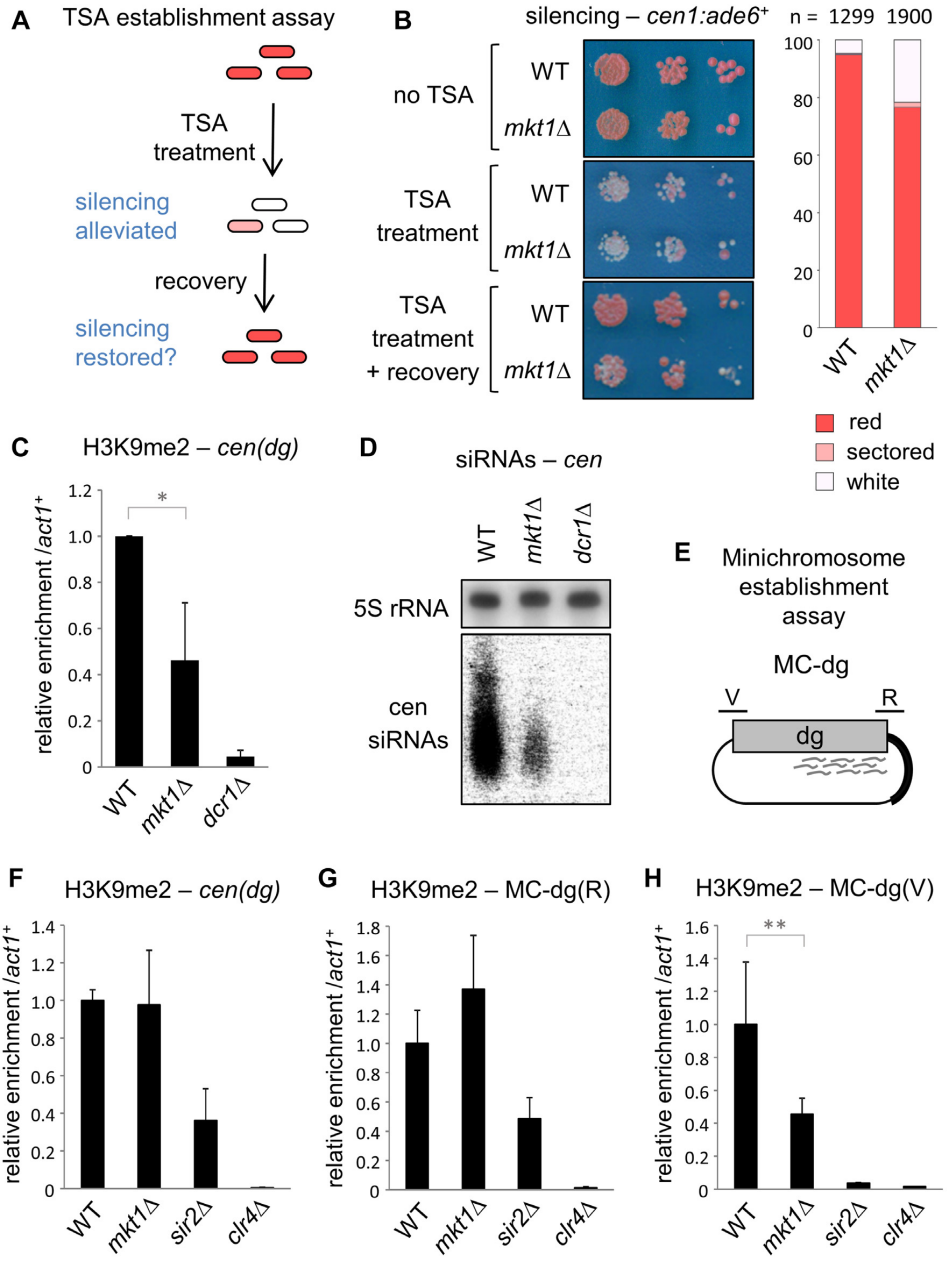
Mkt1 is required for efficient establishment of pericentromeric heterochromatin. (**A**) Schematic representation of the TSA heterochromatin re-establishment assay. Cells carrying the *cen1:ade6^+^* reporter are treated with the HDAC inhibitor trichostatin A (TSA) which causes loss of H3K9me and silencing at *cen1:ade6^+^*, resulting in pink/white colonies on low adenine media. Following a period of recovery from TSA treatment, wild-type cells re-establish silencing resulting in red colonies; continued appearance of pink/white colonies indicates a defect in heterochromatin establishment. (**B**) Assay for silencing of *cen1*:*ade6^+^*. Cells were plated on low adenine media prior to TSA treatment (no TSA), after growth for 10 generations in medium containing 35 μg/ml TSA (TSA treatment), and after growth for a further 10 generations in the absence of TSA (TSA treatment + recovery). The chart shows the frequency of red, white and sectored colonies following TSA treatment and recovery. (**C**) ChIP-qPCR analysis of H3K9me2 levels at *cen(dg)* relative to *act1^+^*, normalised to wild-type, following TSA treatment and recovery. (**D**) Northern analysis of centromeric siRNAs following TSA treatment and recovery (5S rRNA is a loading control). (**E**) Schematic representation of the *MC-dg* plasmid used for the minichromosome establishment assay. The plasmid carries centromere central core sequence plus 5.6 kb of *dg* outer repeat sequence. The locations of primers used to monitor H3K9 methylation over the siRNA-rich region (R) and siRNA void region (V) are indicated. (**F-H**) In cells transformed with *MC-dg*, ChIP-qPCR analysis of H3K9me2 levels at *cen(dg), MC-dg(R) and MC-dg(V)*, relative to *act1^+^*, normalised to wild-type. In each case data are averages of three biological replicates and error bars represent one SD; *P < 0.05, **P < 0.01.

The observations above indicate that loss of *mkt1^+^* affects maintenance of the relatively unstable hairpin-mediated silencing, but not the robust silencing typically seen at constitutive heterochromatic loci. To test whether constitutive loci might be sensitive to loss of Mkt1 in the early stages of heterochromatin assembly, wild-type and *mkt1Δ* cells were treated with trichostatin A (TSA), an HDAC inhibitor that leads to global loss of H3K9 methylation and hence heterochromatic silencing (43). In the case of wild-type cells, a period of recovery following TSA treatment enables re-establishment of silencing, and hence a return to red colony colour in cells carrying the *cen1:ade6^+^* reporter (Figure 3A and B). However, in cells lacking Mkt1, re-establishment of silencing is impaired, as evidenced by a higher frequency of white/pink colonies following the recovery period. Consistent with this, ChIP analyses confirmed that in *mkt1Δ* cells H3K9 methylation failed to recover to the same level as in wild-type cells. Centromeric siRNA levels were also not fully restored in the *mkt1Δ* cells (Figure 3C and D). Similar results were obtained using an alternative, genetic approach in which *clr4^+^* or *rik1^+^* genes were deleted in order to fully erase H3K9 methylation, and then reintroduced by crossing (Supplementary Figure S2). These observations indicate a defect in establishment of silencing and heterochromatin in the absence of *mkt1^+^*.

To investigate this further we employed another, independent assay for *de novo* heterochromatin formation. It has been shown previously that minichromosome plasmids carrying portions of fission yeast centromeric sequence are substrates for *de novo* heterochromatin assembly (4,44). To test if Mkt1 is required for establishment of heterochromatin on a minichromosome, we transformed wild-type and *mkt1Δ* cells with the previously described MC-dg plasmid, which carries 5.6 kb of ‘*dg*’ outer repeat sequence including a portion targeted by abundant siRNAs (Figure 3E). ChIP-qPCR analysis using plasmid-specific primers revealed establishment of H3K9me2 on the minichromosome in wild-type cells, as reported previously. While H3K9me2 levels maintained on endogenous pericentromeric repeats were indistinguishable between WT and *mkt1Δ* cells (Figure 3F), we found reduced levels of H3K9me2 established on the minichromosome in the absence of Mkt1. Interestingly, this effect was seen specifically at the region of the *dg* sequence not associated with siRNAs (siRNA void, V), whereas establishment of H3K9me2 on the region associated with abundant siRNAs (siRNA rich, R) appeared unaffected (Figure 3G and H). This confirms that absence of Mkt1 is associated with reduced efficiency of *de novo* heterochromatin establishment, and suggests a particular defect in spreading of H3K9 methylation beyond sites of nucleation.

### Mkt1 functions in RNAi-mediated post-transcriptional silencing

**Figure 4.**
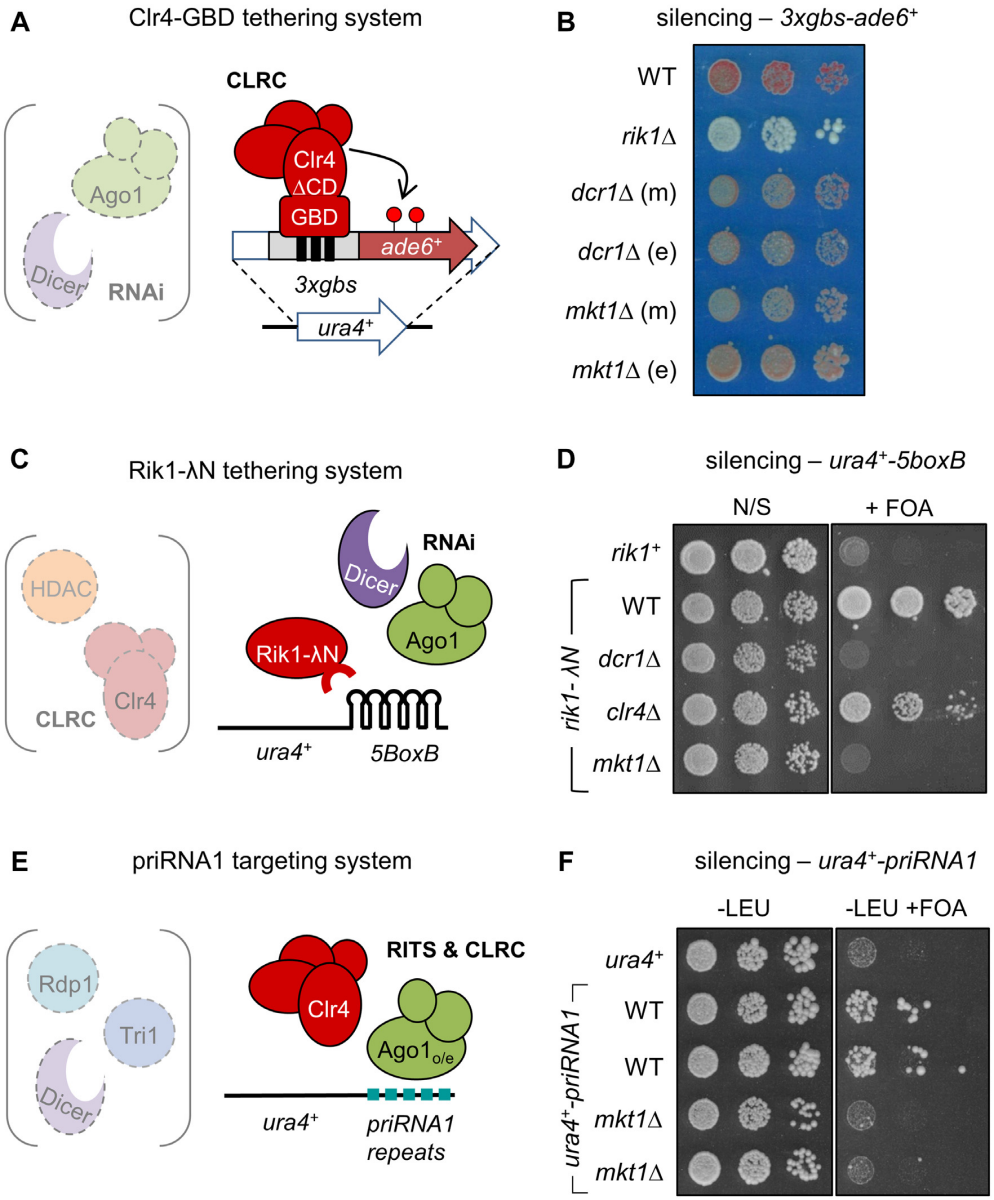
Mkt1 is required for RNAi-mediated post-transcriptional silencing, downstream of siRNA production. (**A**) Schematic representation of the Clr4 tethering system, in which a Clr4-GBD fusion protein is artificially tethered upstream of the *ade6^+^* gene via *gbs* binding sites (*3xgbs-ade6^+^*). This results in silencing that is independent of the RNAi machinery. (**B**) Assay for silencing of the *3xgbs-ade6^+^* reporter: red colonies on low adenine media indicate silencing of *ade6^+^*; failure of silencing results in white colonies. Deletion of the indicated genes before or after bringing together the two components of the silencing system (Clr4-GBD and *3xgbs-ade6^+^*) enabled assessment of establishment (e) or maintenance (m) of silencing, respectively. (**C**) Schematic representation of the Rik1 tethering system, in which a Rik1-N fusion protein is artificially tethered to a *ura4^+^* reporter RNA via five *boxB* binding sites in the 3′UTR (*ura4^+^-5BoxB*). This results in silencing that is independent of the chromatin modification machinery. (**D**) Assay for silencing of the *ura4^+^-5BoxB* reporter: silencing of *ura4^+^* permits growth on media containing 5-FOA; loss of growth on 5-FOA indicates loss of silencing. (**E**) Schematic representation of the priRNA1 silencing system, in which an rRNA-derived small RNA, priRNA1, directs silencing of a *ura4^+^* reporter RNA containing priRNA1 complementary repeat sequences in the 3′UTR (*ura4^+^-priRNA1*). Silencing is dependent on over-expression of Ago1, but independent of the normal siRNA biogenesis machinery. (**F**) Assay for silencing of the *ura4^+^-priRNA1* reporter: growth on media containing 5-FOA indicates silencing of *ura4+* (media lacking leucine is used to maintain selection for Ago1 over-expression).

Heterochromatin assembly in *S. pombe* is characterised by an interdependence between RNAi and H3K9 methylation that makes it difficult to define where in the pathway a particular component acts. To get around this problem and narrow down the function of Mkt1, we employed two systems that were developed to uncouple RNAi from chromatin modification. First, to determine whether *mkt1^+^* is required for H3K9 methylation independently of RNAi, we employed a system in which the H3K9 methyltransferase Clr4 is artificially tethered to chromatin, bypassing the requirement for RNAi-mediated recruitment (45). In this system Clr4 is fused to the Gal4 DNA binding domain (GBD-Clr4), targeting it to a reporter locus comprising three Gal4 binding sites upstream of the *ade6^+^* gene (*3xgbs-ade6^+^*; Figure 4A). In wild-type cells this results in H3K9 methylation and silencing of *ade6^+^* that is dependent on CLRC complex components and other chromatin modifiers, but independent of RNAi (45). By deleting *mkt1^+^* either before or after the establishment of silencing via this system, we were able to test the effect of *mkt1^+^* deletion on either maintenance of pre-existing silencing, or *de novo* establishment of silencing. In contrast to removal of the CLRC subunit Rik1, but akin to absence of the RNAi component Dcr1, deletion of *mkt1^+^* had little effect on either establishment or maintenance of silencing mediated by tethered Clr4 (Figure 4B). This was confirmed by RT-qPCR and ChIP analyses, which indicated that *ade6^+^* transcript and H3K9me2 levels in *mkt1Δ* cells were similar to those in wild-type cells (Supplementary Figure S3). Thus *mkt1^+^* appears to be dispensable for heterochromatin assembly independently of RNAi.

To investigate whether *mkt1^+^* is instead required for RNAi-mediated silencing independently of chromatin modification, we turned to an alternative system in which a Rik1-λN fusion protein is artificially tethered to a modified *ura4^+^* reporter RNA containing five boxB binding sites in the 3′UTR (*ura4^+^-5boxB*; Figure 4C and (46)). As shown previously, tethering of Rik1 results in silencing of the *ura4^+^* reporter that is dependent on RNAi, but independent of Clr4 and other chromatin modification components, i.e. this silencing operates at the post-transcriptional level. Strikingly, we found that deletion of *mkt1^+^*, like deletion of *dcr1^+^*, disrupts maintenance of *ura4^+^* silencing in this system, as indicated by loss of growth on media containing the counter-selective drug 5-FOA (Figure 4D). These observations indicate that *mkt1^+^* is important for RNAi-mediated post-transcriptional silencing.

One other factor has previously been reported to be required specifically for establishment but not maintenance of RNAi-mediated silencing at centromeres in *S. pombe*: a nuclease named Triman (Tri1) that is involved in siRNA biogenesis (32). To test whether Mkt1 might function alongside Tri1 in small RNA biogenesis, we employed another artificial system in which siRNA production is uncoupled from downstream silencing (Figure 4E and (47)). In this system the normal siRNA biogenesis pathway is bypassed, and silencing is instead triggered by an abundant, naturally occurring small RNA (‘priRNA1’) that is derived from rRNA, independently of Dcr1. The reporter is a *ura4^+^* transcript that has been made into a target for these rRNA-derived small RNAs by insertion of complementary sequence into the 3′UTR (*ura4^+^-priRNA1*). Silencing in this system has been shown to be dependent on the RNAi effector Ago1 and downstream chromatin modifiers, but independent of factors involved in normal small RNA biogenesis including Dcr1, Rdp1 and Tri1 (47). We predicted that if Mkt1 functions together with Tri1 in small RNA production, it too would be dispensable for silencing in this system. However, this is not the case: the silencing of the *ura4^+^-priRNA1* reporter seen in wild-type cells was lost upon deletion of Mkt1 (Figure 4F), indicating that Mkt1 acts in the RNAi pathway downstream of small RNA biogenesis.

To confirm that Mkt1 functions in series with RNAi, we also tested whether *mkt1^+^* deletion enhances the heterochromatin defects seen in cells lacking the RNAi component Dcr1. Whereas impairing the function of RNA processing factors Rrp6 or Dhp1 has been shown to exacerbate the defects in RNAi mutants indicating independent, parallel functions (20–22), we found that H3K9me2 levels in an *mkt1Δ dcr1Δ* double mutant are equivalent to those in a *dcr1Δ* single mutant, consistent with these factors acting in the same pathway (Supplementary Figure S3C).

### Mkt1 functions together with Ath1 but not MTREC

The results above point to a role for Mkt1 in post-transcriptional silencing. This is consistent with studies of Mkt1 homologs in *Saccharomyces cerevisiae* and trypanosomes, both of which have also been implicated in post-transcriptional gene regulation, although precise molecular functions remain unclear (48,49). Analyses in *S. cerevisiae* indicate that Mkt1 forms a complex with polyA-binding protein 1-binding protein Pbp1 (49), and this interaction appears conserved in trypanosome (48) and in *S. pombe* (50), where the Pbp1 homolog is named Ath1. Interestingly, in *S. pombe* both Mkt1 and Ath1 have also been found to associate with Mtl1, a core component of the MTREC/NURS complex involved in RNA surveillance (24,25,29). To further investigate Mkt1-interacting proteins we FLAG-tagged Mkt1 at the endogenous locus and performed affinity purification followed by liquid chromatography–tandem mass spectrometry (LC–MS/MS). After Mkt1, the next most abundant protein identified in our purifications was Ath1, consistent with previous observations (Supplementary Table S3). Other interactors included both nuclear and cytoplasmic proteins, consistent with the reported localisation of Mkt1 to both nucleus and cytoplasm (51). While the identified proteins provided little further insight into the molecular function of Mkt1, we did note that several of these Mkt1 interactors were also previously found to interact with Mtl1 and/or other MTREC-associated factors, consistent with Mkt1 functioning as part of a post-transcriptional regulatory network.

**Figure 5.**
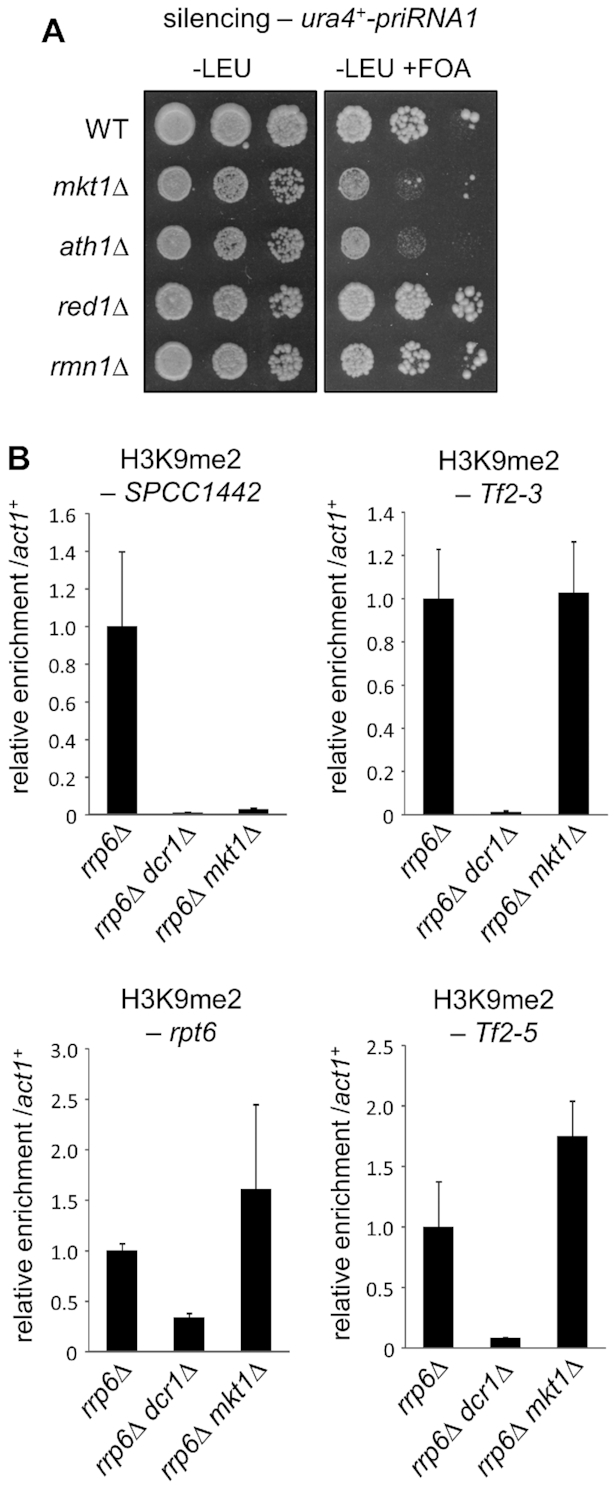
Mkt1 is required for maintenance of H3K9 methylation at specific HOODs. (**A**) Analysis of silencing of the *ura4^+^-priRNA1* reporter by the rRNA-derived small RNA priRNA1, as described in Figure 4. (**B**) ChIP-qPCR analysis of H3K9me2 levels at four previously described HOODs, relative to *act1^+^*, normalised to wild-type. In each case data are averages of three biological replicates and error bars represent one SD.

To investigate whether Mkt1 functions in cooperation with Ath1 and/or MTREC in RNAi mediated silencing, we returned to the priRNA1 silencing assay to test for any defects in silencing in the absence of Ath1, the core MTREC component Red1, or the alternative Mtl1-binding partner Nrl1 (24) (deletion of *mtl1^+^* was not tested as it is an essential gene). Interestingly, deletion of *ath1^+^* resulted in loss of silencing as seen in *mkt1Δ* cells, suggesting that Ath1 and Mkt1 function together in post-transcriptional regulation (Figure 5A). In contrast, silencing was unaffected upon deletion of *red1^+^* or *nrl1^+^*. To further explore any possible functional connection with Mtl1, we investigated the role of a sequence motif (LFXØD) that we noticed in the N-terminus of Mkt1. This motif was shown to be required for interaction of specific adaptor proteins with the exosome-associated helicase Mtr4 (52), which shares significant homology with Mtl1, including in the ‘arch’ region required for interaction with the LFXØD sequence. Although the motif is not conserved in *S. cerevisiae* Mkt1 (Supplementary Figure S4A), we speculated that it could be required for some aspect of Mkt1 function in *S. pombe*, where (unlike in *S. cerevisiae*) RNAi is present. To test this, we replaced the endogenous *mkt1^+^* gene with a version in which the five residues of this motif were mutated to alanines (*mkt1–5A*). However, in contrast to deletion of *mkt1*^+^, the *mkt1–5A* mutant had no effect on priRNA1-mediated silencing (Supplementary Figure S4B), indicating that the LFXØD motif is not required for the silencing function of Mkt1. Together these results suggest that Mkt1 functions together with Ath1 but independently of Mtl1/MTREC to mediate silencing.

The N-terminus of Mkt1 also contains a region with structural similarity to PIN nuclease domains, found in proteins involved in a range of core cellular processes including RNA processing and degradation. Structure and sequence analyses place Mkt1 in the FEN-like class of PIN-like domain proteins, which includes structure-specific nucleases such as FEN-1 and XPG, involved in DNA replication and nucleotide excision repair, respectively (53). However, while the majority FEN-like PIN domains contain six highly conserved active site residues, all of which have been shown to be required for catalytic activity of human FEN2 (53,54), only two of these residues are conserved in the PIN-like domains of Mkt1 in *S. pombe* and *S. cerevisiae* (Supplementary Figure S4A). It therefore appears unlikely that these proteins retain nuclease activity. To explore this further, we replaced the endogenous *mkt1^+^* gene with versions bearing mutations in the two remaining conserved residues linked to catalytic activity: D34A and D201A. Unlike *mkt1^+^* deletion, neither of these point mutants disrupted priRNA1-mediated silencing (Supplementary Figure S4B), further supporting the conclusion that Mkt1 function in silencing is unlikely to involve nuclease activity.

### Mkt1 is required for maintenance of certain facultative heterochromatin domains

In addition to pericentromeric heterochromatin, the RNAi pathway is also required to maintain the small domains of facultative heterochromatin termed HOODs that are detected in the absence of the nuclear exosome component Rrp6, principally at transposons and genes involved in sexual differentiation (23). To investigate whether Mkt1 plays any role in maintenance of these endogenous heterochromatin domains, we constructed an *mkt1Δ rrp6Δ* double mutant strain. Strikingly, ChIP-qPCR analysis of H3K9me2 levels at several well-characterised HOODs revealed locus-specific effects of Mkt1 loss. At the meiotic gene SPCC1442.04c, H3K9me2 was completely abolished in the absence of Mkt1, the same as in *dcr1Δ* cells. In contrast, at two HOODs associated with Tf2 retroelements, Tf2–3 and Tf2–5, as well as at the proteasome gene *rpt6^+^*, H3K9me2 levels were unaffected or even slightly increased upon loss of Mkt1 (Figure 5B). As anticipated, *mkt1^+^* deletion had no effect on silencing at heterochromatin islands that are maintained independently of RNAi (Supplementary Figure S5). Interestingly, while the MTREC subunit Red1 is generally required for maintenance of HOODs, the HOOD that we found to be Mkt1-dependent (SPCC1442.04c), was notable in a previous analysis for being Red1-independent (23). These findings are therefore suggestive of Mkt1 functioning in a parallel pathway to Red1 in the regulation of HOODs. In addition, the highly locus-specific effects of *mkt1^+^* deletion as compared to *dcr1^+^* deletion indicate that the phenotypes seen in cells lacking Mkt1 do not simply reflect a mild defect in RNAi-mediated silencing in general, but rather indicate a specific role for Mkt1 in silencing that is context-dependent.

### Mkt1 is involved in non-coding RNA regulation

Given that Mkt1 is implicated in post-transcriptional regulation, we conducted RNA expression analysis to investigate any changes in gene expression associated with absence of Mkt1. Very few genes were found to have altered expression in *mkt1Δ* cells compared to wild-type (Supplementary Figure S6A and Table S4), indicating that loss of Mkt1 has little impact on global mRNA levels under standard growth conditions. Importantly, no factors implicated in the RNAi pathway showed altered expression, arguing against the possibility that the silencing defect in *mkt1Δ* cells is an indirect effect of altered expression of a pathway component. Interestingly, we noticed that a higher proportion of ncRNAs showed altered expression in the absence of Mkt1 (∼4% of annotated ncRNAs, as compared to 0.6% of mRNAs, Supplementary Figure S6A and Table S5). The majority of affected ncRNAs were upregulated (44 out of 52), suggesting a role for Mkt1 principally in negative regulation of ncRNAs.

To further explore the regulatory targets of Mkt1, we isolated Mkt1-interacting RNAs by a UV crosslinking and immunoprecipitation approach, CRAC (crosslinking and analysis of cDNAs) (55). We observed specific crosslinking of Mkt1 to RNA, supporting its physical RNA association (Supplementary Figure S7A). Sequencing of these Mkt1-bound RNAs revealed specific association of Mkt1 with the 3′UTRs of a subset of mRNAs (Supplementary Figure S7B and Table S6). This is consistent with the known association of Mkt1 with Ath1, the human homolog of which was previously shown to bind to mRNA 3′UTRs (56). We did not observe any clear signal of Mkt1 association with annotated ncRNAs; this may be because these RNAs are generally expressed at low levels. Interestingly however, we did note association of Mkt1 with antisense transcripts derived from the rDNA (Supplementary Figure S6B). Re-examination of our RNA-seq data revealed an increase of ∼3-fold in levels of these unannotated transcripts in *mkt1Δ* cells (Supplementary Figure S6B), consistent with a role for Mkt1 in their regulation.

### Mkt1-mediated regulation of pericentromeric transcripts is important for maintenance of silencing and heterochromatin when transcriptional silencing is impaired

**Figure 6.**
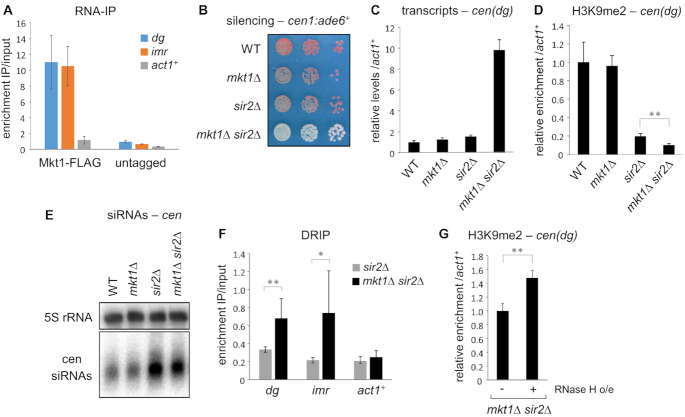
Mkt1 interacts with pericentromeric transcripts and functions in parallel with Sir2 to mediate their silencing. (**A**) RNA immunoprecipitation (RNA-IP) analysis of transcripts associated with FLAG-tagged Mkt1 under native conditions. IP enrichments are shown relative to input. (**B**) Assay for silencing of the *cen1*:*ade6^+^* reporter: red colonies on low adenine media indicate silencing, loss of silencing results in white colonies. (**C**) RT-qPCR analysis of *cen(dg)* transcript levels relative to *act1*^+^, normalized to wild-type. (**D**) ChIP-qPCR analysis of H3K9me2 levels at *cen(dg)* relative to *act1^+^*, normalized to wild-type. (**E**) Northern analysis of centromeric siRNAs (5S rRNA is a loading control). (**F**) DRIP analysis of DNA:RNA hybrid levels at the indicated loci. IP enrichments are shown relative to input. (**G**) ChIP-qPCR analysis of H3K9me2 levels at *cen(dg)* in the *mkt1Δ sir2Δ* strain, with and without RNAse H over-expression. In each case data are averages of three biological replicates and error bars represent one SD; *P < 0.05, **P < 0.01.

If Mkt1 is directly involved in post-transcriptional regulation of pericentromeric transcripts, physical association of Mkt1 with these RNAs would be predicted. Although our CRAC analyses did not detect binding of Mkt1 to non-coding RNAs derived from the pericentromeres, this is not surprising since these transcripts are known to be present at very low levels in wild-type cells. To directly assess whether Mkt1 associates with pericentromeric ncRNAs, we therefore performed RNA-IP analyses in cells lacking the HDAC Sir2, which is involved in transcriptional repression in heterochromatin (4,16). Strikingly, by this method we were able to detect specific enrichment of centromeric transcripts (*dg* and *imr*) in Mkt1-FLAG pull downs, as compared to pull-downs from untagged control cells (Figure 6A). Mkt1 associated more strongly with centromere-derived transcripts than with the control transcript *act1^+^*. These findings demonstrate that Mkt1 physically associates with centromeric transcripts, consistent with it playing a direct role in their regulation.

Our earlier findings as well studies in other systems implicate Mkt1 primarily in post-transcriptional regulation. Why then is it required for efficient establishment but not maintenance of pericentromeric heterochromatin? We reasoned that this may reflect a requirement for post-transcriptional silencing prior to, or as part of, establishment of transcriptional silencing; once robust transcriptional silencing is in place the requirement for post-transcriptional regulation may be lost. We hypothesised that if this were the case, then Mkt1 may also be required for maintenance of heterochromatin in conditions where transcriptional silencing is impaired. To test this we again exploited deletion of the transcriptional silencing factor Sir2. Strikingly, whereas either *mkt1Δ* or *sir2Δ* single mutants have little effect on maintenance of silencing of a *cen1:ade6^+^* reporter (dark pink colonies), *mkt1Δ sir2Δ* double mutants exhibit strong de-repression (white colonies, Figure 6B). RT-qPCR analyses revealed similar synergistic effects on maintenance of silencing of endogenous pericentromeric transcripts, with the *mkt1Δ sir2Δ* double mutant showing a large increase in transcript accumulation compared to either of the two single mutants (Figure 6C). A small but significant further reduction in pericentromeric H3K9 methylation levels in the double mutant was also observed (Figure 6D). The silencing defects occur despite processing of centromeric transcripts into siRNAs being intact, since high levels of siRNAs accumulate in both the *sir2Δ* and *mkt1Δ sir2Δ* strains (Figure 6E). These findings support the conclusion that Mkt1 acts in parallel to Sir2 to maintain pericentromeric silencing. Whereas Sir2 mediates transcriptional silencing, Mkt1 is involved in RNAi-dependent post-transcriptional silencing, and both are required for efficient establishment of heterochromatin.

A possible consequence of impaired post-transcriptional downregulation of pericentromeric transcripts could be increased accumulation of these transcripts on chromatin, possibly resulting in increased DNA:RNA hybrid formation, which has been implicated in defective heterochromatin assembly (57,58). To test whether increased DNA:RNA hybrid accumulation might occur in cells lacking Mkt1, we performed DNA-RNA immunoprecipitation (DRIP). These analyses revealed a modest but reproducible increase in RNA:DNA hybrid accumulation at pericentromeres in *mkt1Δ sir2Δ* double mutant cells, as compared to the *sir2Δ* single mutant (Figure 6F). No such increase was seen at the control locus *act1^+^*. Moreover, over-expression of RNase H in *mkt1Δ sir2Δ* cells led to a small increase in pericentromeric H3K9 methylation, suggesting that suppressing DNA:RNA hybrid accumulation can partially rescue the heterochromatin defect (Figure 6G). These findings are consistent with a model whereby, when transcription at pericentromeres is high (either in an establishment scenario, or when transcriptional silencing is impaired), RNAi- and Mkt1-dependent post-transcriptional silencing promotes heterochromatin assembly at least in part through prevention of over-accumulation of pericentromeric transcripts on chromatin.

## DISCUSSION

Across eukaryotes, mechanisms for maintenance of heterochromatin are typically built around self-reinforcing loops involving positive feedback between histone methylation and DNA methylation and/or small RNAs. Such mechanisms ensure robust propagation of constitutive heterochromatin domains, however less is known about factors and mechanisms involved during the initial establishment of heterochromatin. These may be important during the dynamic assembly of facultative heterochromatin domains, as backup for maintenance mechanisms, as well for establishment of new heterochromatin domains over evolutionary time, such as in response to novel insertions of invasive genetic elements. Here we have identified a novel role for the RNA-associated factor Mkt1 in RNAi-mediated silencing and heterochromatin establishment. We show that Mkt1 associates with pericentromeric transcripts, and is required for efficient establishment of heterochromatin at pericentromeres. Although dispensable for maintenance of pericentromeric heterochromatin, it is required to maintain some facultative heterochromatin domains. Our findings indicate a function for Mkt1 in RNAi-dependent post-transcriptional silencing, and provide evidence that this post-transcriptional silencing is required to facilitate efficient heterochromatin assembly and transcriptional silencing in certain contexts.

Mkt1 homologues have been found across eukaryotes, including fungi and protozoa, although not in plants or animals. Our finding that Mkt1 is required for RNAi-mediated post-transcriptional silencing in *S. pombe* is broadly consistent with studies in other systems that have demonstrated conserved roles for Mkt1 in post-transcriptional regulation. In *S. cerevisiae*, quantitative trait loci (QTL) mapping studies have identified functional associations between Mkt1 sequence variants and several complex phenotypes including tolerance to high temperature, ethanol, and other stresses (59–62), and have implicated Mkt1 in gene regulation at the RNA level (63). While expression studies indicate effects of Mkt1 on RNA stability, studies of the HO mRNA have also produced evidence of Mkt1-mediated regulation at the level of translation (49). Mkt1 is similarly implicated in RNA regulation in trypanosomes, where it interacts with multiple RNA binding proteins and is proposed to function as part of a post-transcriptional regulatory network (48). Together these studies imply a role for Mkt1 in general mRNA metabolism, and our observations are consistent with this also being the case to some extent in *S. pombe*: by CRAC we observed Mkt1 association with the 3′ ends of a small subset of mRNAs. These Mkt1-bound mRNAs were enriched for genes related to small molecule metabolism. However, deletion of *mkt1^+^* has no apparent effect on general fitness under normal growth conditions, and our RNA-seq analyses revealed only a small number of genes with altered expression in *mkt1Δ* cells (and these showed no particular ontology enrichment). Although it remains to be tested, we suspect that Mkt1-mediated mRNA regulation (as opposed to its role in RNAi-directed ncRNA regulation, discussed below) may be important primarily in stress conditions; this would be consistent with the stress sensitivity phenotypes associated with Mkt1 sequence variants in *S. cerevisiae*. Perhaps surprisingly, there was little overlap between the mRNAs found to associate with Mkt1, and those showing altered expression in *mkt1Δ* cells. In accordance with observations made in trypanosomes (48), this likely reflects the fact that Mkt1 functions as part of a regulatory network, and hence the functional outcome of Mkt1 binding is dependent on association of other factors.

In both *S. cerevisiae* and trypanosomes, Mkt1 functions together with its interaction partner Pbp1, a well conserved protein with homologues in higher eukaryotes (Ataxin-2, ATXN2). Our proteomic analyses indicate that Mkt1 also interacts with the Pbp1 homologue, Ath1, in *S. pombe*. Pbp1^Ath1/Ataxin-2^ proteins have been implicated in several different aspects of RNA regulation, and it appears that this regulation can be either positive or negative depending on the associated factors. For example, in *S. cerevisiae*, while Pbp1^Ath1/Ataxin-2^ most commonly seems to be involved in upregulation of gene expression, it is also implicated in negative regulation of ncRNAs, with *pbp1^+^* deletion resulting in increased accumulation of RNA-DNA hybrids at rDNA (64). Moreover, in *Drosophila*, distinct Pbp1^Ath1/Ataxin-2^-containing complexes have been identified that mediate up- and down-regulation of different target RNAs (65). Of particular note, Pbp1^Ath1/Ataxin-2^ exerts negative regulation of a subset of mRNAs via a role in miRNA-mediated silencing in conjunction with the conserved DEAD-box RNA helicase DDX6 (66). Here we provide evidence that Mkt1 and Ath1^Pbp1/Ataxin-2^ function together in siRNA-mediated post-transcriptional silencing in *S. pombe*. In clear parallels to the findings in other systems described above, we observed accumulation of rDNA-derived ncRNAs in the absence of Mkt1, and interestingly, our mass spectrometry analyses also revealed Mkt1 interaction with the *S. pombe* homolog of DDX6, Ste13. Thus our findings suggest that Mkt1 and associated factors may play analogous roles in RNAi-dependent and –independent post-transcriptional silencing in different systems.

Post-transcriptional silencing is the canonical mode of RNAi-mediated regulation, and can occur at multiple levels. In siRNA-mediated silencing in plants and animals, substrate RNAs undergo ‘dicing’ to generate siRNAs, while Argonaute proteins with the requisite catalytic activity subsequently mediate ‘slicing’ of target RNAs based on siRNA sequence complementarity. In the case of miRNAs, incomplete complementarity between small RNA and target disfavours slicing, and instead silencing is achieved primarily through Argonaute-mediated recruitment of other factors that mediate translation repression and RNA degradation (67). In *S. pombe*, the inherent coupling of RNAi to transcriptional silencing in *cis* means the extent and significance of RNAi-mediated post-transcriptional silencing is less clear. Studies using artificial systems, including the Rik1 tethering system developed previously and employed here, have demonstrated that the fission yeast RNAi machinery has the potential to mediate post-transcriptional silencing (46,68). Consistent with this, the *S. pombe* Argonaute protein is competent for slicer activity. However, a recent study revealed that while Ago1 slicer activity is required for siRNA maturation, it is not required for silencing in the priRNA1 silencing system in which normal siRNA biogenesis requirements are bypassed (47). This suggests that either post-transcriptional silencing is not important in this system, or that it requires involvement of additional post-transcriptional regulatory factors, more akin to miRNA-mediated silencing. Here we find that Mkt1 is required for silencing in the priRNA1 system, as well as in the Rik1 tether system that specifically reports on RNAi-mediated post-transcriptional silencing. Our findings, together with evidence from previous studies in other systems, suggest that Mkt1 is a post-transcriptional regulatory factor that can act in conjunction with RNAi to mediate silencing. Since deletion of Mkt1 appears to specifically impede RNAi-mediated post-transcriptional silencing, uncoupling it from RITS-directed recruitment of chromatin modifiers in *cis*, our findings shed light on the functional significance of RNAi-mediated post-transcriptional silencing in *S. pombe*, providing evidence that such silencing does have functional relevance in certain contexts.

We have shown that Mkt1 is involved in establishment, and in some cases maintenance, of RNAi-dependent heterochromatin. Given that Mkt1 appears to be required primarily for RNAi-mediated post-transcriptional silencing, how does its absence impact on heterochromatin integrity? A paradox of systems in which ncRNAs serve as nucleators of heterochromatin in *cis* is that heterochromatin assembly is dependent on transcription of the locus, yet both the process of transcription and the accumulation of heterochromatic transcripts have the potential to interfere with heterochromatisation. We envisage at least two ways in which failure to remove heterochromatic transcripts post-transcriptionally could negatively impact on heterochromatin assembly. First, if these transcripts are allowed to accumulate off chromatin, they could compete with nascent RNAs for binding of RITS and associated factors, potentially reducing the efficiency of silencing in *cis*. Alternatively, if they accumulate on chromatin, the transcripts may hinder access of other factors, or impede heterochromatin assembly in other ways. Several lines of evidence support the latter hypothesis. First, in our minichromosome establishment assay, lack of Mkt1 did not affect nucleation of H3K9 methylation at the siRNA rich region, but did affect its spreading to the siRNA void region. This suggests that the defect was not in RITS-targeting, but rather in the subsequent spreading. Second, two recent studies found that deletion of components of the Ccr4-Not complex impairs subtelomeric heterochromatin (in particular its spreading from nucleation sites) (57,69) and that this is associated with increased retention of RNA on chromatin and formation of RNA:DNA hybrids (57). Third, our analyses suggest that loss of Mkt1 is also associated with an increase in RNA:DNA hybrid accumulation at pericentromeres. We therefore favour a model whereby Mkt1 contributes to removal of heterochromatic transcripts, and when this removal is impaired, transcript accumulation on chromatin can hinder heterochromatin assembly. Whereas the Ccr4-Not complex acts in parallel with RNAi to mediate silencing at telomeres, Mkt1 acts in the same pathway as RNAi for silencing at centromeres, suggesting that these pathways represent alternative means to similar ends, with their relative importance varying between different genomic loci. Such mechanisms to reconcile transcription and chromatin modification may be a common theme in systems where ncRNAs nucleate heterochromatin in *cis*, which occur throughout eukaryotes.

Given that RNAi is required for both establishment and maintenance of heterochromatin at pericentromeres, why is Mkt1 required only for efficient establishment but not for maintenance of pericentromeric heterochromatin? We show here that in the absence of the transcriptional silencing factor Sir2, maintenance of pericentromeric heterochromatin becomes sensitive to loss of Mkt1, suggesting that Mkt1-dependency may relate to transcript levels. In the simplest scenario we envisage that in a maintenance situation where transcriptional silencing is already in place, relatively low levels of pericentromeric transcripts are produced, and post-transcriptional silencing is dispensable. In contrast, during establishment, when transcriptional silencing is not yet imposed, higher levels of pericentromeric transcripts are generated, and hence the need for Mkt1-mediated post-transcriptional transcript clearance is greater. Why Mkt1 is required for maintenance of hairpin-mediated silencing is unclear, but likely relates to the relatively inefficient silencing in this system: H3K9 methylation levels at the target locus are modest compared to those at pericentromeres, and so in the absence of robust transcriptional silencing there may be an ongoing requirement for post-transcriptional regulation. The highly locus-specific requirement for Mkt1 at certain HOODs (SPCC1442.04c) and not others is particularly interesting, indicating that the requirement for Mkt1 is strongly context-dependent. A number of factors could potentially be involved, including levels of transcription, the tendency for specific transcripts to be retained on chromatin, and/or coupling to particular developmental signals. Further work is required to understand the basis of this specificity, but given that the one HOOD that we found to be absolutely Mkt1-dependent was previously noted as Red1-independent, it is tempting to speculate that Mkt1 and Red1 could operate in parallel pathways, both of which cooperate with RNAi at different loci.

Mkt1 joins a growing list of post-transcriptional regulatory factors contributing to heterochromatin integrity at different loci. However, while other factors such as Rrp6 and Dhp1 have been shown to function in parallel to RNAi, our analyses indicate that Mkt1 acts in series with RNAi, unveiling the requirement for RNAi-mediated post-transcriptional silencing in heterochromatin assembly. In the future it will be interesting to investigate the molecular mechanism by which this protein contributes to silencing in *S. pombe*, as well as whether, as appears to be the case in *S. cerevisiae*, Mkt1 plays a role in coordinating gene expression changes in response to stress, which could include modulation of heterochromatin.

## DATA AVAILABILITY

RNA-seq and CRAC datasets have been deposited in the Gene Expression Omnibus under accession numbers GSE135273 and GSE135735, respectively. Mass spectrometry data have been deposited to the ProteomeXchange Consortium via the PRIDE repository with the dataset identifier PXD015484.

## Supplementary Material

gkz1157_Supplemental_File

## Notes

Present address: Francesca Taglini, MRC Human Genetics Unit, Institute of Genetics and Molecular Medicine, University of Edinburgh, Edinburgh, EH4 2XU, UK; Edinburgh Cancer Research Centre, Institute of Genetics and Molecular Medicine, Western General Hospital, Crewe Road, Edinburgh EH4 2XU, UK.
