# Supplementary material for: Mkt1 is required for RNAi-mediated silencing and establishment of heterochromatin in fission yeast
Source: Nucleic Acids Res. 2019 Dec 11;48(3):1239–53. doi: 10.1093/nar/gkz1157 (PMC7026591; doi:10.1093/nar/gkz1157)
Supplement: gkz1157_Supplemental_File [file gkz1157_supplemental_file.pdf]

SUPPLEMENTARY METHODS

Genome-wide genetic screen

The genetic screen was performed using version 2 of the haploid deletion library constructed by the Bioneer Corporation and the Korea Research Institute of Biotechnology and Bioscience (1). For the screen, each of the 3088 G418-resistant deletion strains in the library was crossed to a tester strain bearing the components of the hairpin silencing system (*GFP-HP*:*leu1*$^{+}$*; ade6*$^{+}$*-GFP*$^{+}$*:natR*) plus a recessive cycloheximide resistance allele for selection against diploids (2). Manipulations were carried out using a Singer RoToR colony pinning robot, essentially as described previously (2). First, the library was arrayed in 384 colony format, four colonies per deletion strain, on YES agar containing 100 ug/ml G418. The tester strain was also arrayed in 384 colony format on YES agar containing 100 ug/ml ClonNat. Library and tester stain cells were then combined together on ME plates, and incubated at 25°C for 3 days. The resulting cell/spore mixture was then transferred directly onto selective media (PMG lacking leucine, and supplemented with arginine, histidine and uracil at 100ug/ml; adenine at 6.25 ug/ml [1/15th normal concentration]; and G418, ClonNat and cycloheximide at 100 ug/ml) and incubated at 32°C for 7 days. The plates were then incubated for a further 5 days at 4°C to allow colony colour to develop prior to visual analysis. Strains generating uniformly white colonies, indicative of loss of silencing of *ade6*$^{+}$*-GFP*$^{+}$, were selected. To focus on potential novel pathway components, strains representing factors already known to be required for maintenance of chromatin silencing in *S. pombe* (based on GO annotation on pombase (3)) were discarded. The remaining strains were subject to secondary screening at the molecular level by RT-qPCR analysis of *ade6*$^{+}$*-GFP*$^{+}$ transcript accumulation and ChIP analysis of H3K9me2 levels at the *ade6*$^{+}$*-GFP*$^{+}$ locus. Only one strain displayed significant changes in both *ade6*$^{+}$*-GFP*$^{+}$ transcript and H3K9me2 levels compared to wild-type: a deletion of *mkt1*$^{+}$. An independent *mkt1*Δ deletion strain was generated to validate the findings, and this strain was used for subsequent analyses.

SUPPLEMENTARY REFERENCES

1. Kim, D.-U., Hayles, J., Kim, D., Wood, V., Park, H.-O., Won, M., Yoo, H.-S., Duhig, T., Nam, M., Palmer, G. et al. (2010) Analysis of a genome-wide set of gene deletions in the fission yeast Schizosaccharomyces pombe. *Nat Biotech.*, **28**, 617-623.
2. Roguev, A., Wiren, M., Weissman, J.S. and Krogan, N.J. (2007) High-throughput genetic interaction mapping in the fission yeast Schizosaccharomyces pombe. *Nat. Methods*, **4**, 861-866.
3. Lock, A., Rutherford, K., Harris, M.A., Hayles, J., Oliver, S.G., Bähler, J. and Wood, V. (2018) PomBase 2018: user-driven reimplementation of the fission yeast database provides rapid and intuitive access to diverse, interconnected information. *Nucleic Acids Res.*, **47**, D821-D827.

SUPPLEMENTARY FIGURES

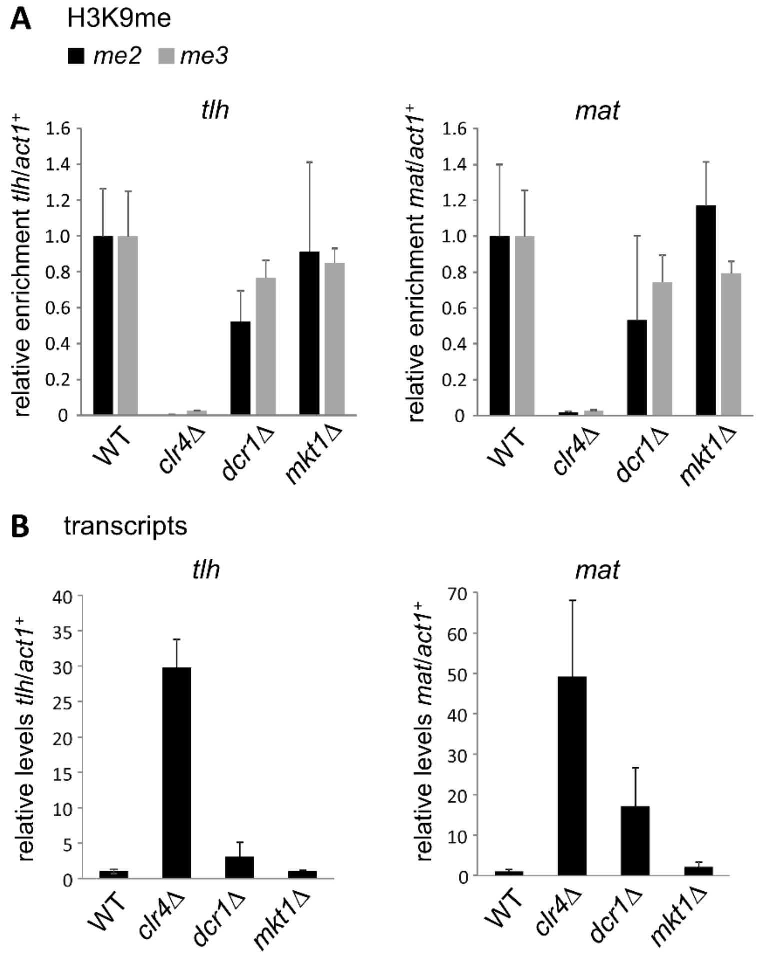

Figure S1. Loss of Mkt1 does not affect maintenance of heterochromatin at telomeres or the silent mating-type locus. (A) ChIP-qPCR analysis of H3K9me2 and H3K9me3 levels at telomeres (*tlh*) or the silent-mating locus (*mat*), relative to *act1*$^{+}$*,* normalised to wild-type. (B) RT-qPCR analysis of levels of non-coding transcripts derived from telomeres (*tlh*) or the silent-mating locus (*mat*), relative to *act1*$^{+}$, normalized to wild-type. In all cases data are averages of 3 biological replicates and error bars represent one SD.

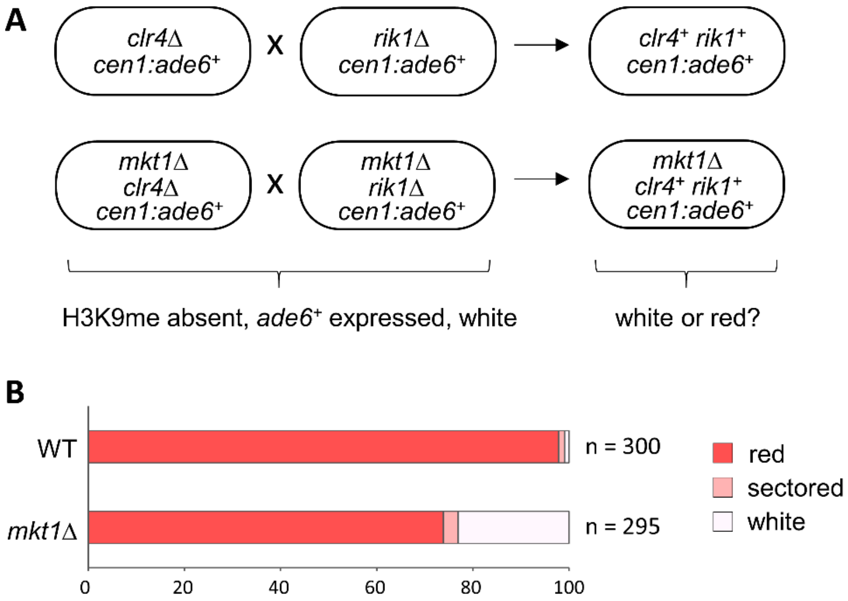

Figure S2. Mkt1 is required for efficient establishment of heterochromatin following removal and reintroduction of CLRC components. (A) Schematic representation of the assay. Cells lacking CLRC complex components Clr4 or Rik1 are devoid of H3K9 methylation resulting in expression of the *cen1:ade6*$^{+}$ reporter and hence white colonies; crossing such cells and selecting *clr4*$^{+}$ *rik1*$^{+}$ progeny allows assessment of heterochromatin establishment, indicated by *cen1:ade6*$^{+}$ silencing and hence red colonies. (B) Quantitative analysis of the frequency of red colonies, indicating heterochromatin establishment, obtained in wild-type and *mkt1*Δ backgrounds.

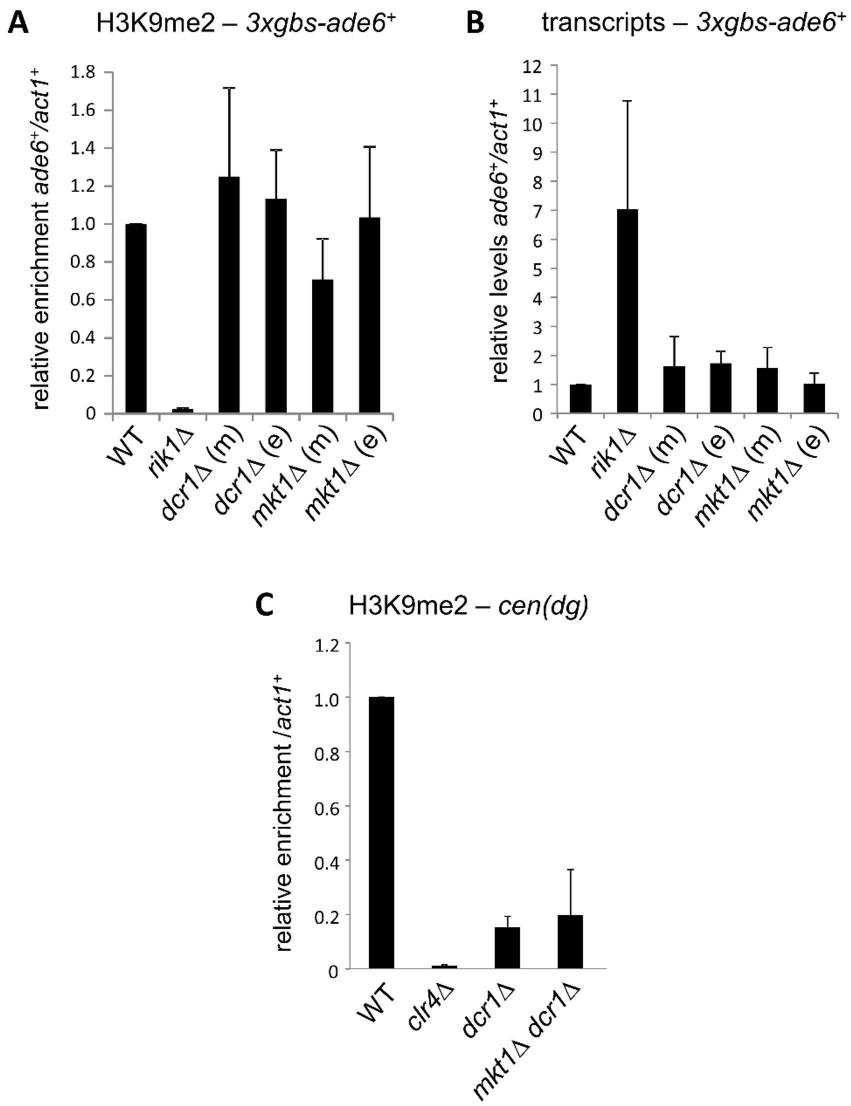

Figure S3. Absence of Mkt1 does not affect silencing mediated by tethered Clr4, and does not enhance the pericentromeric silencing defect associated with absence of Dcr1. (A) ChIP-qPCR analysis of H3K9me2 levels at the *3xgbs-ade6+* locus (targeted by Clr4-GBD) relative to *act1*$^+$*,* normalised to wild-type. (B) RT-qPCR analysis of *3xgbs-ade6*$^+$ transcript levels relative to *act1*$^+$, normalized to wild-type. (C) ChIP-qPCR analysis of H3K9me2 levels at *cen(dg)* relative to *act1*$^+$*,* normalised to wild-type. In all cases data are averages of 3 biological replicates and error bars represent one SD.

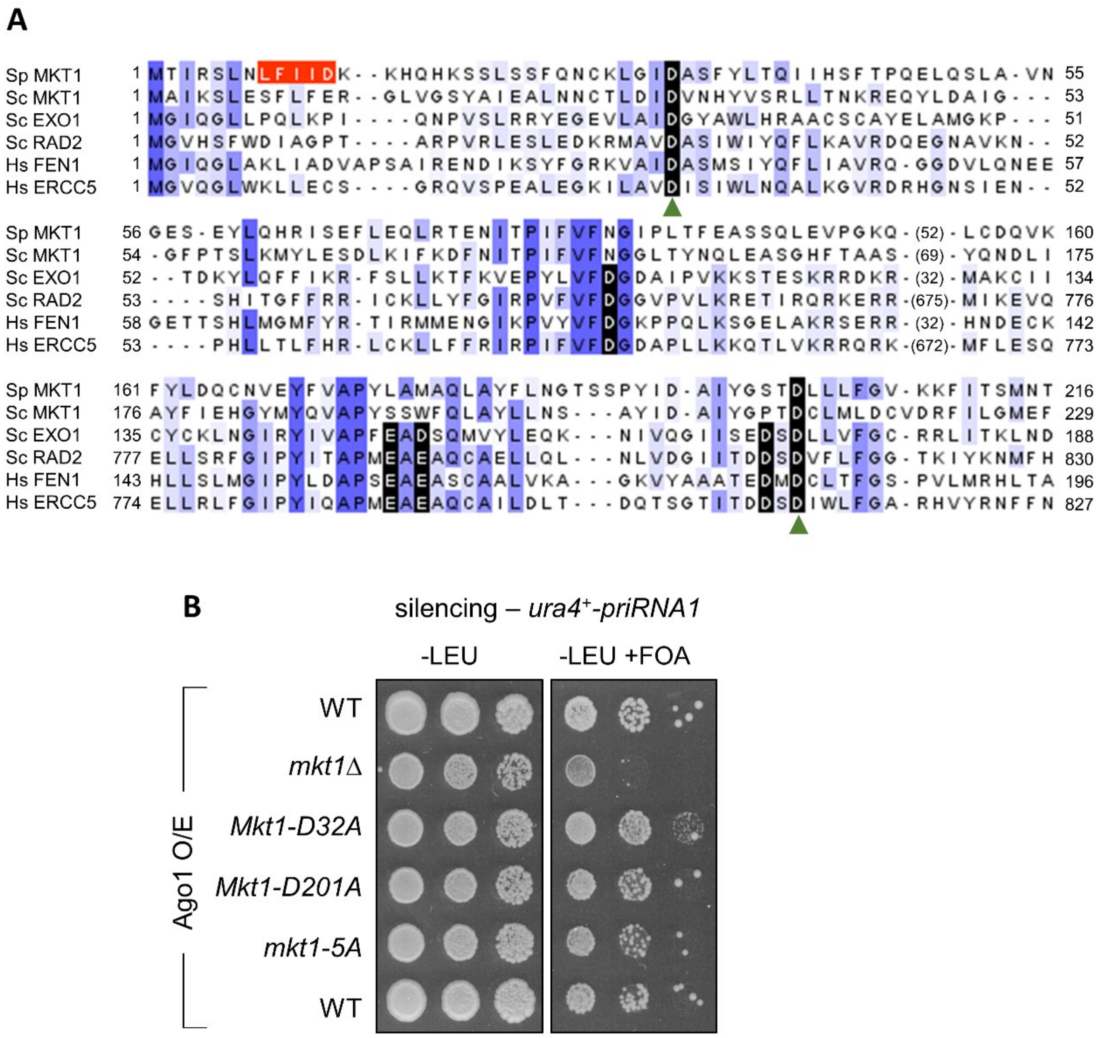

Figure S4. The silencing function of Mkt1 does not depend on either the putative active site residues or the LFXØD motif. (A) Multiple sequence alignment of the conserved core elements of the PIN domain-like region of *S. pombe* Mkt1 and related proteins. The number of excluded residues is shown in parentheses. Sequences aligned using T-Coffee multiple sequence alignment tool: Q9UTN2|MKT1, P40850|MKT1, P39875|EXO1, P07276|RAD2, P39748|FEN1, P28715|ERCC5. Blue shading indicates conservation. Active site residues are highlighted in black; the LFXØD motif is highlighted in red. Green arrows indicate Mkt1 conserved residues D32 and D201. (B) Analysis of priRNA1-mediated silencing of a *ura4*$^{+}$ reporter (*ura4*$^{+}$*-priRNA1*) in the presence of Ago1 over-expression (media lacking leucine is used to maintain selection for the Ago1 over-expression plasmid). Colony growth on media supplemented with 5-fluoroorotic acid (+FOA) indicates silencing of *ura4*$^{+}$.

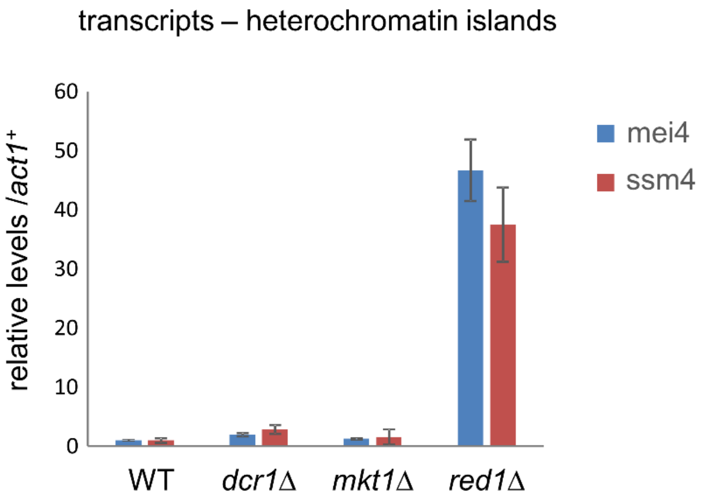

Figure S5. Mkt1 is not required to maintain silencing at heterochromatin islands. RT-qPCR analysis of *mei4*$^{+}$ and *ssm4*$^{+}$ transcript levels, relative to a control transcript *act1*$^{+}$, and normalized to wild-type. Data are averages of 3 biological replicates and error bars represent one SD.

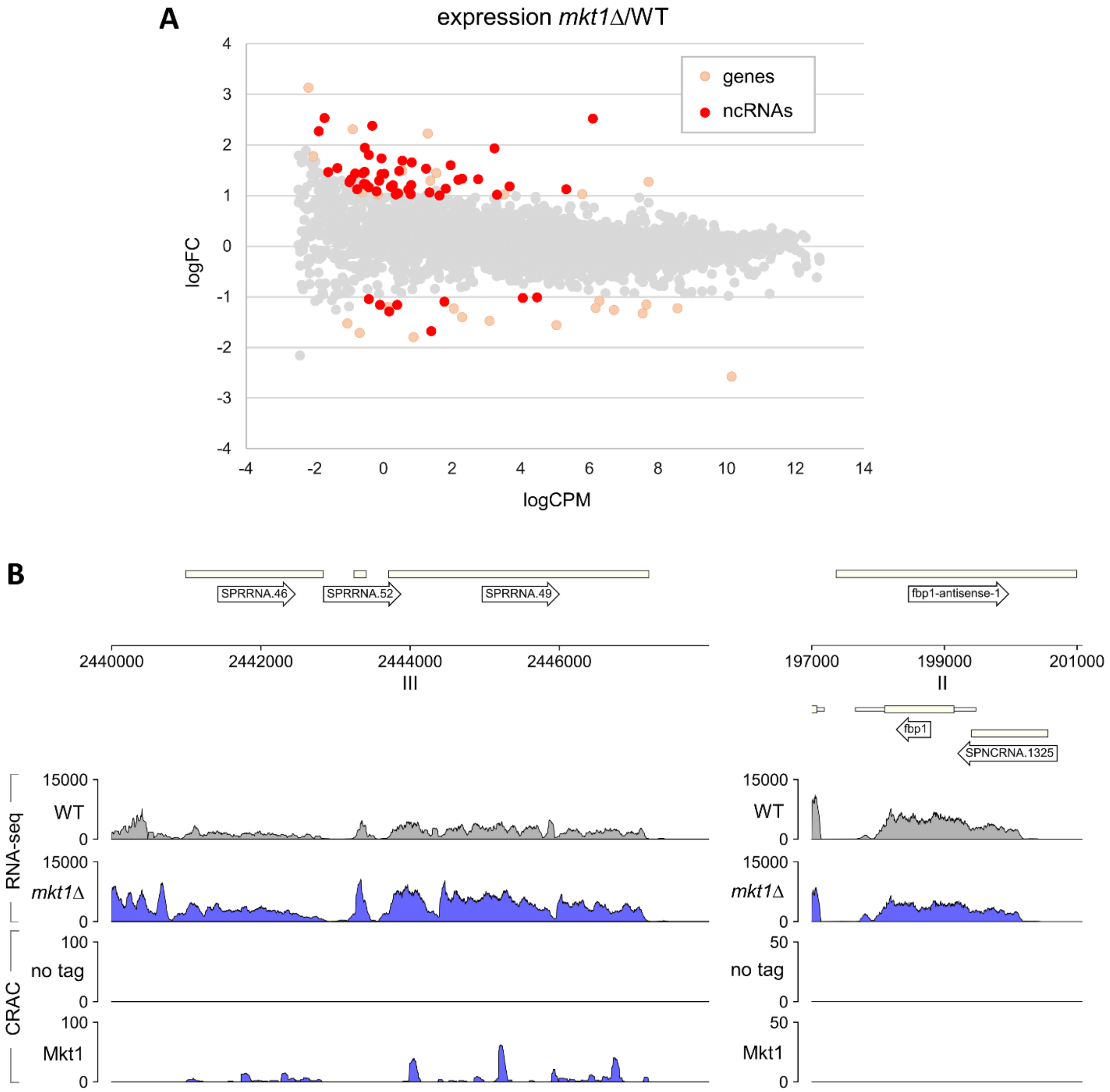

Figure S6. Mkt1 negatively regulates some ncRNAs. (A) Differential expression analysis in *mkt1Δ* cells relative to wild-type. The scatterplot shows the relationship between average expression (counts per million, CPM) and fold-change (FC), both in $\log_2$ scale. Data is shown for all annotated mRNAs and ncRNAs; those showing a significant change of 2-fold or greater are highlighted in orange in the case of mRNAs, and red in the case of ncRNAs. (B) Distribution of RNA-seq and CRAC sequencing reads mapping antisense to the rDNA locus (left); the *fbp*$^{+}$ locus (right) is shown as a control.

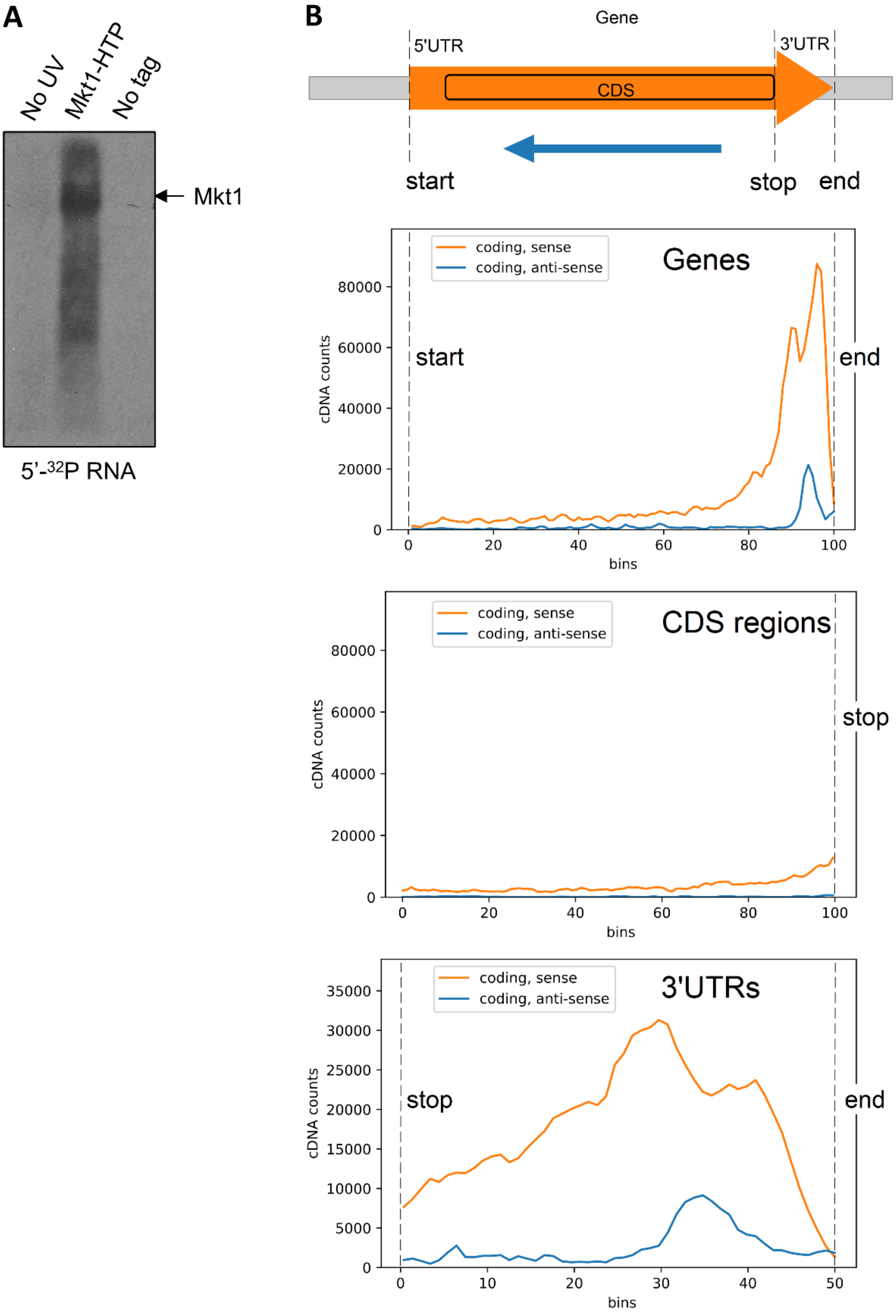

Figure S7. Mkt1 associates with the 3'UTRs of some mRNAs. (A) Autoradiogram showing ^{32}P-labelled RNA cross-linked to Mkt1 tagged with HTP (His6-TEV-ProtA). Purifications from untagged cells, and with no UV crosslinking, serve as negative controls. (B) Metagene analysis of the distribution of Mkt1 CRAC sequencing reads mapping to mRNA transcription units (genes), mRNA coding regions (CDS regions) and mRNA 3'UTRs.

Table S1. List of yeast strains used in this study.

Strain	Genotype	Figure
539	*h- ars1:GFPHP-leu1+ ade6+-GFP-natMX6 cycR leu1-32 ura4-D18*	1
1210	*ade6+-GFP-natMX6 cycR leu1-32 ura4-D18*	1
1150	*h+ mkt1Δ::kanMX6 ars1:GFPHP-leu+ ade6+-GFP-natMX6 cycR leu1-32 ura4-D18*	1
7	*h+ ade6-210 leu1-32 ura4-D18 otr1R(Sph1):ade6+*	2,3,6,S1, S3,S5, S6,S7
114	*h+ clr4Δ:kanMX6 lys1:natMX6 otr1R(Sph1):ade6+ ade6-210 leu1-32 ura4-D18*	2,S1,S3
53	*h+ dcr1Δ::natMX6 otr1R(Sph1):ade6+ ade6-210 ura4-D18*	2,3,S1, S3,S5
2196	*h+ mkt1Δ::natMX6 otr1R(Sph1):ade6+ ade6-210 leu1-32 ura4-D18*	2,3,6,S1, S5,S6
4472	*h+ ade6-704-hygMX6 cc2:his3 ura4-DSE/D18 leu1-32 his3-D1 arg3-D4*	3
4470	*sir2Δ::natMX6 ade6-704 cc2:his3 ura4-D18 leu1-32 his3-D1 arg3-D4*	3
4471	*h90 clr4Δ::LEU2 ade6-704-natMX6 cc2:his3 ura4-DSE/D18 leu1-32 his3-D1*	3
4506	*mkt1Δ::natMX6 ade6-704-hygMX6 cc2:his3 ura4-DSE/D18 leu1-32 his3-D1*	3
196	*GBD-clr4-ΔCD<-1kb->hygMX6 ura4::3xgbs-ade6+ ade6-DN/N leu1-32*	4,S3
431	*rik1Δ::kanMX6 GBD-clr4-ΔCD<-1kb->hphMX6 ura4::3xgbs-ade6+ ade6-DN/N leu1-32 (maintenance)*	4,S3
199	*dcr1Δ::natMX6 GBD-clr4-ΔCD<-1kb->hphMX6 ura4::3xgbs-ade6+ ade6-DN/N leu1-32 (maintenance)*	4,S3
2606	*dcr1Δ::natMX6 GBD-clr4-ΔCD<-1kb->hphMX6 ura4::3xgbs-ade6+ ade6-DN/N leu1-32 (establishment)*	4,S3
2608	*mkt1Δ::kanMX6 GBD-clr4-ΔCD<-1kb->hphMX6 ura4::3xgbs-ade6+ ade6-DN/N leu1-32 (maintenance)*	4,S3
2600	*mkt1Δ::kanMX6 GBD-clr4-ΔCD<-1kb->hphMX6 ura4::3xgbs-ade6+ ade6-DN/N leu1-32 (establishment)*	4,S3
708	*h- ura4+:5BoxB-hygMX6*	4
566	*h- ura4+:5BoxB-hygMX6 rik1+-λN-kanMX6*	4
707	*h- ura4+:5BoxB-hygMX6 rik1+-λN-kanMX6 dcr1Δ::natMX6*	4
3074	*ura4+:5BoxB-hygMX6 rik1+-λN-kanMX6 clr4Δ::natMX6*	4
2509	*ura4+:5BoxB-hygMX6 rik1+-λN-kanMX6 mkt1Δ::natMX6*	4
3294	*ura4+:kanMX6 pREP1-Pnmt1-3xflag-ago1 leu1-32 ade6-210*	4
3295	*ura4+:6xpriRNA1-kanMX6 pREP1-Pnmt1-3xflag-ago1 leu1-32 ade6-210*	4,5,S4
3296	*mkt1Δ::natMX6 ura4+:6xpriRNA1-kanMX6 pREP1-Pnmt1-3xflag-ago1 leu1-32 ade6-210*	4,5,S4
5214	*ath1Δ::natMX6 ura4+:6xpriRNA1-kanMX6 pREP1-Pnmt1-3xflag-ago1 leu1-32 ade6-210*	5
5218	*red1Δ::natMX6ura4+:6xpriRNA1-kanMX6 pREP1-Pnmt1-3xflag-ago1 leu1-32 ade6-210*	5
5220	*rmn1Δ::natMX6 ura4+:6xpriRNA1-kanMX6pREP1-Pnmt1-3xflag-ago1 leu1-32 ade6-210*	5
1621	*h+ rrp6Δ::kanMX6 ade6-210 ura4-D18 leu1-32*	5
2505	*dcr1Δ::natMX6 rrp6Δ::kanMX6 otr1R(Sph1):ade6+ ade6-210 leu1-32 ura4-D18*	5
2233	*mkt1Δ::natMX6 rrp6Δ::kanMX6 otr1R(Sph1):ade6+ ade6-210 leu1-32 ura4-D18*	5
140	*h- sir2Δ::natMX6 otr1R(Sph1):ade6+ ade6-210 leu1-32 ura4D18*	6
4375	*mkt1Δ::natMX6 sir2Δ::ura4+ otr1R(Sph1):ade6+ ade6-210 leu1-32 ura4D18*	6
4512	*Mkt1-3xFlag-natMX6 sir2Δ::ura4+ otr1R(Sph1):ade6+ ade6-210 leu1-32 ura4-D18*	6
5618	*mkt1Δ::NatMX6 sir2Δ::ura4+ otr1R(Sph1):ade6+ ade6-210 leu1-32 ura4D18 pRep1-empty*	6

5620	*mkt1Δ::NatMX6 sir2Δ::ura4+ otr1R(Sph1):ade6+ ade6-210 leu1-32 ura4D18 pRep1-rnh201-GFP*	6
2175	*h- mkt1Δ::kanMX6 clr4Δ::ura4+ otr1R(Sph1):ade6+ ade6-210 leu1-32 ura4-D18*	S2
2231	*h+ mkt1Δ::kanMX6 rik1Δ::hygMX6 otr1R(Sph1):ade6+ ade6-210 leu1-32 ura4-D18*	S2
2164	*h+ clr4Δ::ura4+ otr1R(Sph1):ade6+ ade6-210 leu1-32 ura4-D18*	S2
2597	*h- rik1Δ::hygMX6 otr1R(Sph1):ade6+ ade6-210 leu1-32 ura4-D18*	S2
2264	*h+ mkt1Δ::kanMX6 dcr1Δ::natMX6 otr1R(Sph1):ade6+ ade6-210 leu1-32 ura4-D18*	S3
5222	*mkt1-5A:natMX6 ura4+:6xpriRNA1-kanMX6 pREP1-Pnmt1-3xflag-ago1+ leu1-32 ade6-210*	S4
5224	*mkt1-D32A:natMX6 ura4+:6xpriRNA1-kanMX6 pREP1-Pnmt1-3xflag-ago1+ leu1-32 ade6-210*	S4
5226	*mkt1-D201A:natMX6 ura4+:6xpriRNA1-kanMX6 pREP1-Pnmt1-3xflag-ago1+ leu1-32 ade6-210*	S4
5228	*mkt1+:natMX6 ura4+:6xpriRNA1-kanMX6 pREP1-Pnmt1-3xflag-ago1+ leu1-32 ade6-210*	S4
2889	*h90 red1Δ::kanMX6 leu1-32 ura4D18 ade6-210*	S5
3189	*h+ mkt1-HTP-KanMX6 otr1R(Sph1):ade6+ ade6-210 leu1-32 ura4D18*	S6,S7

Table S2. List of primers used in this study.

Primer	Sequence	Use
q_dg_F	AATTGTGGTGGTGTGGTAATAC	qPCR
q_dg_R	GGGTTCATCGTTTCCATTCAG	qPCR
q_imr_F	CTAATGCGGAGTAAGGCTAATC	qPCR
q_imr_R	TGGACAGAATGGATGGATATTG	qPCR
q_ade6_F	ATGCTTATCCTACAACTGAGACC	qPCR
q_ade6_R	TGAATTGAGAAGGGAAGACGAG	qPCR
q_GFPHP_F	CAACCATTACCTGTCCACACA	qPCR
q_GFPHP_R	AAAAAGTTCCAACACACCTGA	qPCR
q_ura4_F	CGTGGTCTCTTGCTTTGG	qPCR
q_ura4_R	GTAGTCGCTTTGAAGGTTAGG	qPCR
q_act_F	GGTTTCGCTGGAGATGATG	qPCR
q_act_R	ATACCACGCTTGCTTTGAG	qPCR
q_MC-dgV_94	AATACGACTCACTATAGGGCGAATTG	qPCR
q_MC-dgV_92	ATCGTCACAGTTTACAAATTCGGT	qPCR
q_MC-dgR_89	ATCATTCAGAAAATCACCGGAGCAAT	qPCR
q_MC-dgR_91	TCGCCCTAAAAGTAAACGGTAAGC	qPCR
q_RPT6_207F	GAGAATCCATTCGAGGTCCA	qPCR
q_RPT6_207R	AATGCAAACATACCGGCTTC	qPCR
q_SPCC1442.04_110F	CCAAACTCTGTTGTTGCAGAAG	qPCR
q_SPCC1442.04_110R	GATTCCTCAAGGTCGTTATCCC	qPCR
q_Tf2-5_F	AGGGCTGTAAGACAATAGTGAAG	qPCR
q_Tf2-5_R	AGGTCGGTAGTCGATATACCAT	qPCR
q_Tf2-3_F	GTTCACAAATCGGTTTCCAG	qPCR
q_Tf2-3_R	GCGTGCACTATAGTGAACAATC	qPCR
q_mat_F	GTCCGAGGCAATACAACTTTGG	qPCR
q_mat_R	GGTTGACAGTAGGAGATATTTACAG	qPCR
q_tlh1_F	GGATAAGCCAATCATCGTTGAG	qPCR
q_tlh1_R	GTAGTTGACGCTCCTTGGAAG	qPCR
q_mei4_F	AAAAGCGACCTTCAAGCAAA	qPCR
q_mei4_R	TTGCATCGTTTGAGACTTCG	qPCR
q_ssm4_F	AACAGCTAAAGACCGCAAGG	qPCR
q_ssm4_R	TCTCCTTGCAGGCAAAGGTC	qPCR
IK8	ATTCCTTTCTGAACCTCTCTGTTAT	*cen* siRNA probe
IK9	TTTGATGCCCATGTTCATTCCACTTG	*cen* siRNA probe
IK10	GGGAGTACATCATTCCTACTTCGATA	*cen* siRNA probe
GFP8	AAGGAGAAGAACTTTTCACTGGAGTTGTCCCAATTCTTG	GFP siRNA probe
GFP48	TGAATTAGATGGTGATGTTAATGGGCACAAATTTTCTGT	GFP siRNA probe
GFP88	AGTGGAGAGGGTGAAGGTGATGCAACATACGGAAAACT	GFP siRNA probe

GFP128	CCCTTAAATTTATTTGCACTACTGGAAAACTACCTGTTCC	GFP siRNA probe
5S rRNA	GTATGGCCGTAGACACCTAGT	5S rRNA probe

Table S3. List of Mkt1-interacting proteins identified by affinity purification and mass spectrometry, based on identification of two or more peptides in each of two biological replicates, and a total peptide count at least 10-fold greater than in the negative control IPs (also show are previously reported interactions of the identified proteins with MTREC-associated factors Mtl1, Red1, Red5, or Pab2).

Systematic ID	Name	Product description	Total peptide count		Reported Interactors
			Mkt1	control	
SPAC139.01c	mkt1	XP-G family nuclease Mkt1	127	2	Mtl1
SPBC21B10.03c	ath1	ataxin-2 homolog	96	1	Mtl1, Red5
SPCC1223.06	tea1	cell end marker Tea1	62	0	
SPAC20G8.05c	cdc15	F-BAR domain protein Cdc15	57	1	Red1
SPAC18G6.07c	mra1	rRNA (pseudouridine) methyltransferase Mra1	39	0	
SPBC16H5.12c		DUF2433 metallo phosphatase superfamily conserved fungal protein	32	3	Mtl1, Red1, Red5
SPBC776.09	ste13	ATP-dependent RNA helicase Ste13	30	0	Pab2, Red5
SPBC776.08c	utp22	small-subunit processome, UTP-C complex subunit Utp22 (predicted)	26	0	Mtl1
SPAC16E8.15	tif45	translation initiation factor eIF4E, 4F complex subunit	13	0	
SPBC776.17	rrp7	rRNA processing protein Rrp7 (predicted)	12	0	
SPBC17F3.01c	rga5	RhoGAP, GTPase activating protein Rga5	11	0	
SPBC1706.01	tea4	tip elongation aberrant protein Tea4	4	0	

Table S4. List of genes showing altered expression in *mkt1*∆ cells (change of ≥2 fold based on three biological replicates).

	Systematic ID	Name	Product description	logFC
upregulated	SPMIT.10	atp9	F1-FO ATP synthase subunit 9 (predicted)	3.130
	SPBPB10D8.04c		transmembrane transporter (predicted)	2.311
	SPCC417.15		dubious	2.224
	SPBC32H8.15		dubious	1.770
	SPCC18.19c	ost5	oligosaccharyltransferase complex zeta subunit Ost5 (predict)	1.490
	SPAC8E11.08c		dubious	1.443
	SPBCPT2R1.08c	tlh2	RecQ type DNA helicase Tlh1	1.293
	SPCC330.03c		NADPH-hemoprotein reductase (predicted)	1.268
	SPAPB1A11.01	mfc1	prospore membrane copper transmembrane transporter Mfc1	1.074
	SPBC23G7.13c		plasma membrane urea transmembrane transporter (predict)	1.061
	SPAC513.03	mfm2	M-factor precursor Mfm2	1.031
	SPBC1271.08c		Schizosaccharomyces pombe specific protein	1.024
	SPBPB8B6.04c	grt1	transcription factor Grt1 (predicted)	1.024
	SPMIT.07	atp6	F1-FO ATP synthase subunit 6 (predicted)	1.020
downregulated	SPBC1289.16c	cao2	copper amine oxidase-like protein Cao2	-1.082
	SPBC337.16	cho1	phosphatidyl-N-dimethylethanolamine N-methyltransferase	-1.156
	SPBC1289.14		adducin (predicted)	-1.207
	SPAC23H3.15c		Scizosaccharomyces specific protein	-1.224
	SPAC343.12	rds1	ferritin related conserved fungal protein	-1.229
	SPAPB1A11.02		esterase/lipase (predicted)	-1.234
	SPAC9E9.09c	atd1	aldehyde dehydrogenase (predicted)	-1.265
	SPBC56F2.11	met6	homoserine O-acetyltransferase Met6	-1.329
	SPAC3G6.07		Schizosaccharomyces specific protein	-1.405
	SPAC27D7.09c		But2 family protein, similar to cell surface molecules	-1.477
	SPAC869.07c	mel1	alpha-galactosidase, melibiase	-1.529
	SPAC4H3.08		3-hydroxyacyl-CoA dehydrogenase (predicted)	-1.562
	SPAC869.06c	hry1	HHE domain cation binding protein (predicted)	-1.716
	SPAC869.09		Con-6 family conserved fungal protein	-1.800
	SPBC14F5.05c	sam1	S-adenosylmethionine synthetase	-2.577

Table S5. List of ncRNAs showing altered expression in *mkt1*∆ cells (change of ≥2 fold based on three biological replicates).

	Systematic ID	Product description	logFC
upregulated	SPNCRNA.175	non-coding RNA (predicted)	2.531
	SPNCRNA.130	intergenic RNA Omt3	2.517
	SPNCRNA.1348	intergenic RNA (predicted)	2.379
	SPNCRNA.281	non-coding RNA (predicted)	2.269
	SPNCRNA.22	non-coding RNA (predicted)	1.940
	SPNCRNA.1124	intergenic RNA (predicted)	1.929
	SPNCRNA.462	non-coding RNA (predicted)	1.800
	SPNCRNA.1342	intergenic RNA (predicted)	1.732
	SPNCRNA.513	non-coding RNA (predicted)	1.690
	SPNCRNA.218	non-coding RNA (predicted)	1.651
	SPNCRNA.316	intergenic RNA (predicted)	1.598
	SPNCRNA.1387	intergenic RNA (predicted)	1.543
	SPNCRNA.1076	antisense RNA (predicted)	1.528
	SPNCRNA.29	non-coding RNA (predicted)	1.486
	SPNCRNA.346	non-coding RNA (predicted)	1.466
	SPNCRNA.278	non-coding RNA (predicted)	1.460
	SPNCRNA.1299	intergenic RNA (predicted)	1.437
	SPNCRNA.1148	intergenic RNA (predicted)	1.432
	SPNCRNA.1493	intergenic RNA (predicted)	1.427
	SPNCRNA.604	intergenic RNA (predicted)	1.422
	SPNCRNA.1251	intergenic RNA (predicted)	1.329
	SPNCRNA.427	non-coding RNA (predicted)	1.323
	SPNCRNA.264	non-coding RNA (predicted)	1.322
	SPNCRNA.1141	intergenic RNA (predicted)	1.307
	SPNCRNA.915	intergenic RNA (predicted)	1.292
	SPNCRNA.253	non-coding RNA (predicted)	1.266
	SPNCRNA.200	non-coding RNA (predicted)	1.227
	SPNCRNA.1283	antisense RNA (predicted)	1.219
	SPNCRNA.188	non-coding RNA (predicted)	1.210
	SPNCRNA.1108	antisense RNA (predicted)	1.203
	SPNCRNA.1023	antisense RNA (predicted)	1.180
	SPNCRNA.1314	intergenic RNA (predicted)	1.173
	SPNCRNA.1262	intergenic RNA (predicted)	1.160
	SPNCRNA.1248	intergenic RNA (predicted)	1.132
	SPNCRNA.187	non-coding RNA (predicted)	1.122
	SPNCRNA.32	non-coding RNA (predicted)	1.121
	SPNCRNA.239	intergenic RNA (predicted)	1.116
	SPNCRNA.154	non-coding RNA (predicted)	1.085
	SPNCRNA.928	antisense RNA Meu16	1.059
	SPNCRNA.1506	intergenic RNA (predicted)	1.042

	SPNCRNA.1310	intergenic RNA (predicted)	1.025
	SPNCRNA.1107	intergenic RNA (predicted)	1.020
	SPNCRNA.1487	antisense RNA (predicted)	1.013
	SPNCRNA.1650	intergenic RNA (predicted)	1.000
**downregulated**	SPNCRNA.960	antisense RNA (predicted)	-1.011
	SPNCRNA.687	antisense RNA (predicted)	-1.023
	SPNCRNA.848	intergenic RNA (predicted)	-1.049
	SPNCRNA.1659	antisense RNA (predicted)	-1.100
	SPNCRNA.845	intergenic RNA (predicted)	-1.161
	SPNCRNA.1131	antisense RNA (predicted)	-1.161
	SPNCRNA.1219	intergenic RNA (predicted)	-1.289
	SPNCRNA.899	intergenic RNA (predicted)	-1.679

Table S6. List of Mkt1-bound mRNAs identified by CRAC (≥10 counts in each of two biological replicates).

Systematic ID	Name	Product description	Counts
SPBC14F5.05c	sam1	S-adenosylmethionine synthetase	2993
SPAC9.09	met26	homocysteine methyltransferase Met26	2406
SPBC8D2.18c		adenosylhomocysteinase (predicted)	1393
SPAC24C9.12c	shm1	serine hydroxymethyltransferase Shm1 (predicted)	1334
SPCC364.07	ser3	D-3 phosphoglycerate dehydrogenase Ser3 (predicted)	974
SPAC13G6.06c	gcv2	glycine cleavage complex subunit P (predicted)	568
SPAC31G5.14	gcv1	glycine decarboxylase T subunit (predicted)	416
SPBP19A11.01	gcv3	glycine decarboxylase complex subunit H (predicted)	357
SPBC428.11	met17	homocysteine synthase Met17	308
SPBC26H8.03	cho2	phosphatidylethanolamine N-methyltransferase Cho2	268
SPCPB16A4.03c	ade10	bifunctional IMP cyclohydrolase/phosphoribosyl-aminoimidazolecarboxamideformyltransferase	230
SPBC839.16	thf1	C1-5,6,7,8-tetrahydrofolate (THF) synthase, trifunctional enzyme Thf1	184
SPBC32F12.11	tdh1	glyceraldehyde-3-phosphate dehydrogenase Tdh1	174
SPBC56F2.11	met6	homoserine O-acetyltransferase Met6	159
SPBC14F5.04c	pgk1	phosphoglycerate kinase Pgk1 (predicted)	123
SPAC1F8.07c	pdc101	pyruvate decarboxylase (predicted)	91
SPBC8D2.15	lip5	mitochondrial lipoic acid synthetase Lip5 (predicted)	87
SPAC27E2.11c		Schizosaccharomyces specific protein	68
SPCC13B11.01	adh1	alcohol dehydrogenase Adh1	60
SPBC19C2.07	fba1	fructose-bisphosphate aldolase Fba1	59
SPCC1442.12	pps1	CDP-diacylglycerol-serine O-phosphatidyltransferase Pps1	57
SPCC1223.02	nmt1	4-amino-5-hydroxymethyl-2-methylpyrimidine phosphate synthase Nmt1	47
SPBP4G3.02	pho1	acid phosphatase Pho1	43
SPAC56F8.10	met9	methylenetetrahydrofolate reductase Met9	39
SPAC1705.03c	ecm33	extracellular leucine-rich repeat domain, receptor L domain-like Ecm33	36
SPAC25G10.03	zip1	transcription factor Zip1	35
SPCC417.08	tef3	translation elongation factor eEF3	31
SPAC29B12.04	snz1	pyridoxine biosynthesis protein	30
SPAC56F8.16	esc1	transcription factor Esc1 (predicted)	29
SPBC16H5.02	pfk1	6-phosphofructokinase pfk1	29
SPBC15C4.04c		amino acid transmembrane transporter (predicted)	29
SPBC26H8.01	thi2	thiazole biosynthetic enzyme	28
SPBC14C8.02	tim44	TIM23 translocase complex subunit Tim44 (predicted)	28
SPAC29A4.02c		translation elongation factor EF-1 gamma subunit	28
SPBC428.02c	eca39	branched chain amino acid aminotransferase Eca39	25
SPCC553.10		conserved fungal cell surface protein, Kre9/Knh1 family (predicted)	25
